# Solid-Phase Synthesis of Azole-Comprising Peptidomimetics and Coordination of a Designed Analog to Zn^2+^

**DOI:** 10.3390/molecules23051035

**Published:** 2018-04-28

**Authors:** Aanchal Mohan, Allyson H. M. Koh, Gregory Gate, Anna L. Calkins, Kyra N. McComas, Amelia A. Fuller

**Affiliations:** Department of Chemistry & Biochemistry, Santa Clara University, Santa Clara, CA 95053, USA; mohanaanchal1@gmail.com (A.M.); allysonkoh21@gmail.com (A.H.M.K.); gate@ucsb.edu (G.G.); acalkins@scu.edu (A.L.C.); sparky6578@msn.com (K.N.M.)

**Keywords:** peptidomimetic, oligoamide, heterocycle, metal coordination

## Abstract

Peptidomimetics that can coordinate transition metals have a variety of potential applications as catalysts, sensors, or materials. A new modular peptidomimetic scaffold, the “azole peptoid”, is introduced here. We report methods for the solid-phase synthesis of eleven examples of trimeric *N*-substituted oligoamides that include oxazole- or thiazole-functionalized backbones. The products prepared comprise a diversity of functionality, including a metal-coordinating terpyridine group. The modular synthetic approach enables ready preparation of analogs for specific applications. To highlight a potential use of this new synthetic scaffold, a trimeric azole peptoid functionalized with a terpyridine residue was prepared and studied. The characteristic 2:1 ligand:metal binding of this terpyridine-functionalized azole peptoid to Zn^2+^ in aqueous solution was observed. These studies introduce azole peptoids as a useful class of biomimetic molecules for further study and application.

## 1. Introduction

One function of natural biopolymers that researchers have sought to recapitulate with modular, bio-inspired oligoamide scaffolds is the ability to coordinate transition metals in aqueous solution. Metal-binding peptidomimetics have potential application as sensors [1,2] or catalysts [3,4], for example. Naturally-occurring molecules, including peptides and peptide-derived natural products, frequently feature well-defined three-dimensional structures that display ligands in a specific arrangement to coordinate to metals. Synthetic oligoamides (i.e., peptidomimetics) offer similar capabilities to display functionality in a spatially controlled manner [5,6,7,8]. Non-natural peptidomimetics have the added advantage that well-studied metal-coordinating ligands not found in biomolecules (e.g., phenanthroline, bipyridine, hydroxyquinoline, or terpyridine) can also be appended [9,10].

Azole metal-coordinating groups are commonly found in peptide-derived natural products [11]. For example, a number of azole-rich, macrocyclic peptide-derived marine natural products have been shown to bind to transition metals, including Cu^2+^, Ag^+^, and Zn^2+^ [12]. In the solid state structures of the copper complex with ascidiacyclamide and the silver complex with westiellamide, the azoles participate as coordinating groups [12,13]. There is interest in the preparation of analogs of this natural product group for a variety of uses, and most examples are macrocycles [14,15,16]. Nonetheless, syntheses frequently cannot easily accommodate the preparation of natural product analogs that include non-natural ligands. The inclusion of high-affinity non-natural ligands may allow for the discovery of synthetically tractable *linear* azole-rich oligoamides that bind to transition metals.

**Figure 1:**
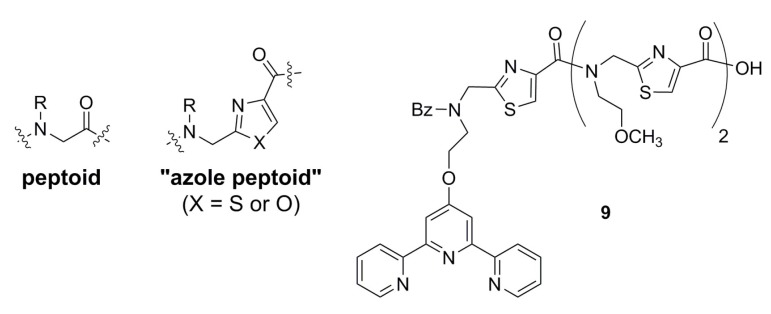
Examples of *N*-substituted oligoamide scaffolds, peptoids and “azole peptoids” described in this work, and the Zn^2+^-coordinating thiazole peptoid analog **9**.

An attractive design for a new oligoamide biomimetic scaffold capable of transition metal binding would preserve the azole-rich features of metal-coordinating natural products and simultaneously accommodate the installation of diverse non-natural ligands with high affinity for metals. Of the available sequence-specific oligoamide scaffolds, peptoids (*N*-substituted glycine oligomers, Figure 1) [17,18] are an especially useful starting point for design of a new peptidomimetic. Their efficient and modular synthesis enables a wide diversity of functionality to be displayed on the *N*-substitutent. Examples of peptoids that leverage the organized display of *N*-substituent functional groups to coordinate to metals have been reported [1,4,10,19,20,21,22,23]. Changing the oligo-glycine backbone unit to insert azoles between the methylene and carbonyl carbon would provide a strategy to prepare peptidomimetics that more closely replicate the metal-coordinating, azole-rich natural product structures. Additionally, this strategy could be compatible with introduction of non-natural ligands as *N*-substitutents. Recently, the inclusion of individual azoles (thiazole, oxazole) into peptoid backbones was reported [24], but an oligoamide with an all-azole backbone (“azole peptoids”, Figure 1) is an unreported scaffold. The metal affinity of azole-comprising peptoids such as **9**, studied here, has also not yet been evaluated. More broadly, the inclusion of azoles into oligoamide backbones is of interest to modulate various physicochemical features of oligomers, including their solubility, polarity, conformational heterogeneity, and hydrogen-bond accepting capabilities [24].

We hypothesized that sequence-specific azole peptoids that display a range of *N*-substituents, including metal-coordinating groups, would be synthetically accessible via modifications to the solid-phase peptoid submonomer synthesis. Here, we report the preparation of eleven examples of diverse trimeric azole peptoids, showcasing the flexibility of the synthesis of this new peptidomimetic. Additionally, we report coordination of the biologically relevant metal Zn^2+^ by terpyridine-functionalized thiazole peptoid **9** (Figure 1) in aqueous buffer. 

## 2. Results

### 2.1. Synthesis of Trimeric Azole Peptoids

**Scheme 1:**
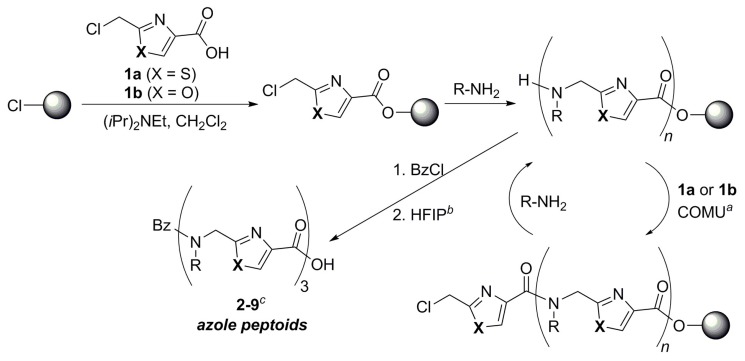
Synthesis of trimeric azole peptoids on solid support. *^a^* 1-[(1-(cyano-2-ethoxy-2-oxoethylidene-aminooxy) dimethylaminomorpholino)] uronium hexafluorophosphate. *^b^* 1,1,1,3,3,3-hexafluoro-2-propanol. *^c^* R group structures are detailed in Table 1.

*N*-substituted oligoamides with an all-azole backbone (“azole peptoids”) have not yet been reported, and we first sought to optimize conditions for their sequence-specific synthesis on solid support (Scheme 1). The commonly-used peptoid submonomer synthesis [25], wherein individual monomers are introduced by iterating bromoacetylation and amine displacement steps, has been modified to allow introduction of individual azole units into a longer oligoglycine backbone by replacing bromoacetic acid with a functionalized azole, **1** [24]. In the reported work, a large excess of **1** (10 equivalents) is used to introduce this backbone modification at the desired position in the peptoid, and both this reaction and the subsequent amine displacement of the chloride are promoted by microwave heating. Because **1** is prepared by a three-step synthesis (see Supplementary Materials), we wished to conserve the amount of **1** needed for the preparation of azole peptoids. Additionally, we sought methods that eliminated the reported microwave heating.

To identify the optimal conditions for the synthesis of azole peptoids, we undertook the synthesis of the trimer-length azole peptoid **2a** (see structure in Table 1), which is capped at the *N*-terminus with a benzoyl group. Synthesis of analogous *N*-substituted oligoamides, arylopeptoids was recently reported; an aromatic ring is inserted into the oligoglycine backbone [26]. We reasoned that the conditions for the preparation of arylopeptoids, which use modest excesses of the acylating reagent and no microwave heating, would be an excellent starting point for the efficient preparation of their counterparts containing azoles. 2-Chlorotrityl chloride resin was found to be suitable for the preparation of arylopeptoids and was used here as well. The progress of two critical synthetic steps in the synthesis of azole peptoid **2a** was monitored using the chloranil test: the displacement of the terminal chloride with 4-(2-aminoethyl)morpholine, and the acylation of the intermediate secondary amine with **1a**. The purities of crude products were assessed by analysis of reverse-phase high-performance liquid chromatography (RP-HPLC) data and compared to identify optimal reaction conditions. The amine displacement reaction was more efficient when heated, as expected. By comparing a small panel of reagents that mediate the *N*-acylation reaction, we identified conditions that allow efficient reaction at room temperature with fewer equivalents of **1a** or **1b** than the reported procedure for the incorporation of **1a** and **1b** into peptoids [24]. We found that using 2.9 equivalents of 1-[(1-(cyano-2-ethoxy-2-oxoethylidene-aminooxy) dimethylaminomorpholino)] uronium hexafluorophosphate (COMU) and just 2.5 equivalents of the custom-synthesized reagent **1a** in the acylation reactions provided **2a** in 85% crude purity and 79% crude yield (Table 1). We evaluated other reagents to promote amide bond formation, including 1-[bis(dimethylamino)methylene]-1*H*-1,2,3-triazolo[4,5-b]pyridinium 3-oxido hexafluorophosphate (HATU) and bis-trichloromethylcarbonate (BTC), but these did not substantially improve the crude yields or purity of **2a**. Purification of **2a** was subsequently effected by preparative RP-HPLC.

With optimal reaction conditions identified, we prepared and purified by RP-HPLC several other examples that comprise an assortment of functionality appended to the azole peptoid scaffold (Table 1). Acids **1a** and **1b** were used to prepare both thiazole and oxazole peptoid trimers, respectively. Reactions proceeded with comparably reliable reactivity for both heterocycles. Varying the primary amines used in the synthesis had more impact on overall yield and purities. However, a diversity of amine substituents was successfully introduced as appended azole peptoid side chains; neutral polar moieties (compounds **2**–**4** and **7**–**9**), nonpolar groups (**5**, **6**, **8**), and charged polar groups (**7**) can all be successfully installed. For the synthesis of **7**, the pendant amine functional group was protected as a *t*-butylcarbamate, and the protecting group was removed concomitant with cleavage of the molecule from the resin using 95% TFA. Notably, we demonstrated with thiazole peptoid **9**, which includes the terpyridine group, that well-studied metal-coordinating groups can be successfully installed. Disappointingly, the incorporation of the sterically demanding amines, such as α-methylbenzylamine, provided negligible yields of the desired product **10**; acylation of the sterically encumbered amine was problematic.

### 2.2. Metal Coordination of **9** in Aqueous Buffer

**Figure 2:**
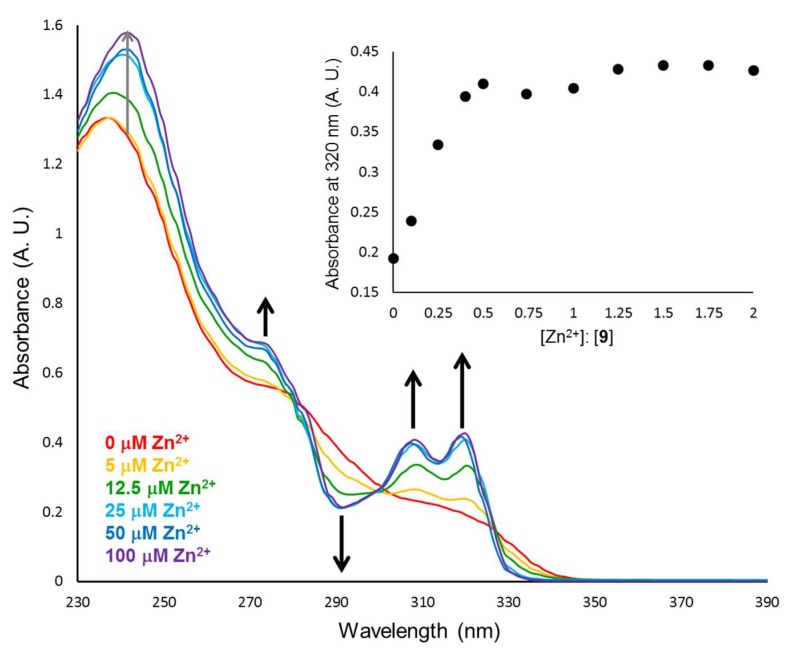
UV-Vis spectra of 50 μM **9** in the presence of varied concentrations of ZnCl_2_ in 10 mM Tris buffer, pH 7.5. Arrows highlight spectral changes with increasing concentrations of Zn^2+^. The inset shows the change in absorbance at 320 nm as a function of the [Zn^2+^]:[**9**] ratio.

Given the successful synthesis of **9**, which includes the terpyridine metal-coordinating ligand in the *N*-terminal position, we explored the potential for **9** to coordinate to the biologically important metal Zn^2+^. We monitored UV spectral changes to an aqueous solution of **9** as Zn^2+^ was titrated in (Figure 2). In the absence of metal, **9** has a maximum at 237 nm and broad shoulders at around 280 and 310 nm. As metal was added to the solution, new spectral peaks emerged at 270, 309, and 320 nm. A plot of the absorbance at the 320 nm maximum as a function of molar ratio of **9**:Zn^2+^ was generated to determine the ratio of the **9**-Zn^2+^ interaction (Figure 2 inset). The spectral changes at 320 nm plateau at [Zn^2+^]:[**9**] = 0.5; one Zn^2+^ is coordinating to two molecules of **9**. This plot looks similar when monitored at other wavelengths (data not shown). To ensure that the terpyridine functionality in **9** is essential for interaction with Zn^2+^, we collected UV spectra of an aqueous solution of **3a** in the presence of increasing concentrations of Zn^2+^. In the absence of the terpyridine coordinating group, no spectral changes were observed upon addition of Zn^2+^ (see Supplementary Materials).

## 3. Discussion

The synthetic methods for azole peptoids optimized here have several advantages. Critically, the methods developed in this work conserve azole reagent **1**, which must be synthesized. The elimination of microwave-promoted synthesis steps previously reported for azole-functionalized peptoids is especially important for the preparation of examples that may coordinate to metals. Many potentially metal-coordinating *N*-substituents include nucleophilic functionality that can undergo unwanted side reactions in the synthesis of peptoids or related oligomers [27]. Additionally, the conditions used for introduction of side chain functionality accommodate appending some nucleophilic side chains that are commonly problematic when used for synthesis of peptoids. For example, the nucleophilic nitrogen in the morpholine ring of 4-(2-aminoethyl)morpholine presents challenges when used as a peptoid submonomer [28]. However, it is an excellent submonomer for the synthesis of azole peptoids **2**. Both the additional atoms of the azole in the peptoid backbone and the reduced reactivity of the chloromethyl group reduce the side reaction that is problematic in the synthesis of oligoglycine analogs.

Applying these synthetic methods, we have accessed a variety of analogs of a new biomimetic oligoamide. Differentially functionalized azole peptoids **7**–**9** highlight the modularity of the approach and the ability to install functionality sequence-specifically for custom applications. An attractive feature of this scaffold is that either **7** or **8** could be readily elaborated via chemoselective reactions with the amine or alkyne, respectively. We anticipate that adaptation of these methods by others will provide synthetic entry to custom azole peptoids that emulate motifs found in natural products or pharmaceuticals. Additionally, azole peptoids with varied lengths can be readily accessed, or combinatorial synthesis of azole peptoid libraries could be undertaken. Use of azole peptoids in screening campaigns could yield exciting applications for this new class of bio-inspired oligoamides.

The binding data obtained for **9** to Zn^2+^ closely matches those observed for homoleptic Zn^2+^-terpyridine complexes investigated by Würthner and co-workers [29]. As was observed in those studies, the lack of curvature in the binding titration plot (Figure 2 inset) precludes the determination of binding constants; the Zn^2+^-terpyridine affinity (K_a_) is estimated at >10^8^ M^−1^ [29]. Moreover, consistent with previous observations, the UV spectra do not change with addition of excess metal ion. The spectral changes observed reflect changes to the conformation of the terpyridine group. The coordination to Zn^2+^ enforces a *cis-cis* configuration of the three pyridine rings [30]. Because of the similarities between our data and these precedents, we speculate that the Zn(**9**)_2_^2+^ complex likewise adopts a distorted octahedral complex [29]. Ongoing studies will evaluate the relative importance of the presence of a high-affinity ligand such as terpyridine and the scaffold flexibility to metal coordination. Indeed, linear azole peptoids show temperature-induced changes to their ^1^H-NMR spectra, confirming their conformational heterogeneity (see Supplementary Materials). Future studies will pursue the ability of more highly-structured oligomers based on the azole peptoid scaffold, including macrocycles, to bind metals.

In summary, we have introduced a new class of peptidomimetic, the azole peptoid, and have demonstrated one potential application of these molecules for coordinating to transition metals in water. The similarities between the azole peptoids and azole-rich natural products make it an attractive scaffold for a range of uses. We anticipate that the straightforward, flexible methods reported here for the synthesis of diversely functionalized azole peptoid analogs will facilitate access to custom-functionalized analogs for further study. Ongoing work in our laboratory will continue to explore relationships between the structures of azole peptoid examples and their capacity to coordinate transitions metals.

## 4. Materials and Methods

### 4.1. General Experimental Information

Compounds **1a** and **1b** were prepared as described in the literature, and their spectral features were identical to those reported [24]. 2-(2,2′:6′,2′′-Terpyridine-4′-yloxyl)ethylamine was also prepared according to literature procedures, and its spectral features were identical to those reported [9]. 2-Chlorotrityl chloride resin was purchased from ChemPep. All other reagents and solvents were purchased from commercial sources and used without further purification. ^1^H and ^13^C-NMR spectra were recorded on a 400 MHz spectrometer using a 5-mm high-resolution direct-detection probe. Chemical shifts (δ) are reported in parts per million (ppm) and are referenced to residual proton in the deuterated solvent, and spectra were acquired at room temperature for compound characterization. Owing to the presence of multiple rotameric states as detailed in the text, all ^1^H-NMR spectra show poor signal dispersion, and peaks appear as complex multiplets. ^13^C-NMR spectra also show broadened and multiple peaks for carbons attributed to different rotamers. The molar masses of purified peptoids were confirmed by electrospray mass spectrometry in positive ion mode. High-resolution mass spectral data were acquired using electrospray ionization and a TOF detector in positive ion mode.

### 4.2. Reverse-Phase High-Performance Liquid Chromatography (RP-HPLC)

The crude purities of the compounds prepared were assessed by analytical RP-HPLC, and products were purified by semi-preparative RP-HPLC. Compounds were eluted from an AAPPTec Spirit Peptide C18 column (5 μM, 0.46 cm × 15 cm for analytical or 5 μM, 10.0 mm × 25 cm for semi-preparative) using a linear gradient of methanol (solvent B) in 0.1% aqueous TFA (solvent A) at 0.75 mL/min (analytical) or 3 mL/min (semi-preparative) flow rate. Peaks eluted were detected by absorbance at 220 nm, and data were visualized with EZChrom software. Chromatograms of both crude and purified compounds are shown in the Supplementary Materials. Purified compounds were isolated by lyophilization to afford white powders.

### 4.3. General Procedure for Synthesis of N-Benzoylated Azole Peptoid Trimers

2-Chlorotrityl chloride resin (137 mg, 1.1 mmol/g, 0.15 mmol) was added to a glass peptide synthesis vessel fitted with a coarse glass frit and a Teflon stopcock, rinsed with CH_2_Cl_2_ (2 × 2 mL), then swelled for five minutes in 2 mL CH_2_Cl_2_, and drained. In a separate vial, CH_2_Cl_2_ (1.3 mL), **1** (32 mg **1a** or 29 mg **1b**, 0.18 mmol, 1.2 equiv), and *N*,*N*-diisopropylethylamine (DIEA) (0.126 mL, 0.72 mmol, 4.8 equiv) were mixed and then added to the synthesis vessel. The mixture was agitated at room temperature for 1 h, drained, and washed with CH_2_Cl_2_ (3 × 2 mL) then DMSO (3 × 2 mL). A solution of the appropriate primary amine (3 mL, 2 M in DMSO, 20 equivalents) was added to the reaction vessel, and the reaction mixture was heated in a sand bath at 50 °C for 1 h. The resin was drained and washed with DMSO (3 × 2 mL), and then NMP (3 × 2 mL). A chloranil test was performed to confirm the presence of a secondary amine (beads turned blue/green).

In a separate vial, **1** (68 mg **1a** or 61 mg **1b**, 0.38 mmol, 2.5 equiv), NMP (0.38 mL), COMU (187 mg, 0.44 mmol, 2.9 equiv), and DIEA (0.157 mL, 0.90 mmol, 6 equiv) were mixed, and the resulting bright red solution was agitated for 5 min before being added to the reaction vessel. The mixture was then agitated for an additional 20 min at room temperature, drained, and washed with NMP (5 × 2 mL) and DMSO (3 × 2 mL). A chloranil test was performed to confirm the absence of a secondary amine (beads remained colorless). The addition of primary amine, washing, then acylation with **1a** or **1b** was iterated to generate trimers. Following the third and final primary amine addition, the resultant secondary amine was reacted with benzoyl chloride (0.060 mL, 0.52 mmol, 3.5 equiv) in CH_2_Cl_2_ (0.52 mL) and DIEA (0.181 mL, 1.04 mmol, 6.9 equiv). The mixture was agitated for 15 min at room temperature, drained, and washed with CH_2_Cl_2_-DIEA 4:1 (3 × 1 mL), CH_2_Cl_2_ (3 × 2 mL), NMP (3 × 2 mL), and CH_2_Cl_2_ (3 × 2 mL).

A 1 mL mixture of 1,1,1,3,3,3-hexafluoro-2-propanol/CH_2_Cl_2_ (1:4) was added to the resin, effecting a color change to bright red, and the mixture was agitated for 1 h at room temperature. The solution was then collected by filtration and combined with CH_2_Cl_2_ washes of the resin (5 × 2 mL). The collected solution was concentrated via rotary evaporation. The crude residue was dissolved in acetonitrile and water, frozen, and lyophilized prior to analysis.

### 4.4. Data for Azole Peptoids Prepared

#### 4.4.1. Thiazole Peptoid Trimer **2a**

The general procedure was followed, using **1a** and 4-(2-aminoethyl)morpholine. Following purification by RP-HPLC using 20–50% gradient of solvent B in solvent A, 80 mg (61%) **2a** were isolated as a white powder. ^1^H-NMR (400 MHz, CD_3_CN) δ 12.71–10.33 (br s, 1H), 8.60–7.74 (m, 3H), 7.74–7.03 (m, 5H), 6.07–4.45 (m, 7H), 4.44–2.42 (m, 35H); ^13^C-NMR (100 MHz, CD_3_CN) δ 172.6, 169.8, 167.2, 165.6, 164.0163.8, 163.3, 162.4, 160.7, 160.3, 160.0, 159.6, 148.3, 147.8, 146.8, 134.6, 134.5, 130.0, 128.9, 128.2, 128.1, 127.9, 127.5, 127.4, 126.4, 120.6, 117.7, 114.8, 111.9, 63.3, 63.0, 60.5, 54.0, 53.9, 53.7, 51.9, 51.8, 50.6, 50.5, 50.0, 47.7, 47.2, 43.5, 42.9, 42.0, 41.8, 41.4, 40.0, 29.0, 27.1; HRMS (ESI-TOF) *m*/*z* calculated for [C_40_H_52_N_9_O_8_S_3_]^+^ 881.302, found 881.303.

#### 4.4.2. Oxazole Peptoid Trimer **2b**

The general procedure was followed, using **1b** and 4-(2-aminoethyl)morpholine. Following purification by RP-HPLC using 10–50% gradient of solvent B in solvent A, 43 mg (33%) **2b** were isolated as a white powder. ^1^H-NMR (400 MHz, CD_3_CN) δ 11.98–9.64 (br s, 1H), 8.45–8.09 (m, 3H), 7.62–7.27 (m, 5H), 5.40–4.27 (m, 13H), 4.27–2.75 (m, 30H); ^13^C-NMR (100 MHz, CD_3_CN) δ 162.4, 160.6, 160.3, 159.9, 145.6, 145.3, 144.7, 135.3, 134.5, 129.9, 128.2, 126.3, 117.9, 115.0, 63.4, 63.3, 63.0, 54.4, 54.3, 53.9, 53.7, 53.5, 53.3, 52.2, 51.8, 51.7, 48.6, 46.4, 46.0, 42.5, 42.0, 40.0, 29.6, 29.0; HRMS (ESI-TOF) *m*/*z* calculated for [C_40_H_52_N_9_O_11_ + H]^+^ 833.371, found 833.371.

#### 4.4.3. Thiazole Peptoid Trimer **3a**

The general procedure was followed, using **1a** and 2-methoxyethylamine. Following purification by RP-HPLC using 55–80% gradient of solvent B in solvent A, 18 mg (13%) **3a** were isolated as a white powder. ^1^H-NMR (400 MHz, CD_3_CN) δ 8.19–7.81 (m, 3H), 7.46–7.05 (m, 5H), 5.39–4.50 (m, 6H), 3.96–2.80 (m, 22H); ^13^C-NMR (100 MHz, CD_3_CN) δ 172.6, 170.7, 168.4, 167.5, 166.9, 166.8, 165.5, 163.6, 163.2, 162.6, 160.7, 160.4, 160.0, 148.9, 148.0, 147.2, 134.5, 130.0, 128.3, 128.0, 126.9, 126.5, 126.2, 120.9, 118.0, 115.0, 112.1, 78.11, 73.4, 71.6, 71.2, 70.0, 69.7, 63.2, 63.0, 57.8, 57.7, 54.0, 53.6, 51.7, 50.2, 49.0, 48.8, 48.6, 48.3, 47.5, 46.2, 43.6, 41.5, 39.5; HRMS (ESI-TOF) *m*/*z* calculated for [C_31_H_37_N_6_O_8_S_3_]^+^ 716.176, found 716.178.

#### 4.4.4. Oxazole Peptoid Trimer **3b**

The general procedure was followed, using **1b** and 2-methoxyethylamine. Following purification by RP-HPLC using 40–80% gradient of solvent B in solvent A, 31 mg (31%) **3b** were isolated as a white powder. ^1^H-NMR (400 MHz, CD_3_CN) δ 8.38–8.08 (m, 3H), 7.53–7.22 (m, 5H), 5.41–4.42 (m, 7H), 4.21–2.94 (m, 29H); ^13^C-NMR (100 MHz, CD_3_CN) δ 163.3, 162.7, 162.4, 161.5, 160.11, 159.7, 145.9, 145.1, 137.2, 136.9, 133.9, 130.7, 129.5, 127.9, 121.0, 115.3, 72.6, 71.3, 71.2, 59.1, 50.2, 59.9, 49.7, 48.1, 47.8, 47.7, 46.4, 45.0, 43.2, 30.4; HRMS (ESI-TOF) *m*/*z* calculated for [C_31_H_37_N_6_O_11_]^+^ 668.244, found 668.241.

#### 4.4.5. Thiazole Peptoid Trimer **4a**

The general procedure was followed, using **1a** and 3-(aminomethyl)pyridine. Following purification by RP-HPLC using 25–30% gradient of solvent B in solvent A, 62 mg (51%) **4a** were isolated as a white powder. ^1^H-NMR (400 MHz, CD_3_OD) δ 9.16–7.78 (m, 15H), 7.58–7.08 (m, 5H), 5.63–4.51 (m, 12H, overlapping with HOD peak); ^13^C-NMR (100 MHz, CD_3_OD) δ 174.5, 169.8, 167.9, 167.6, 165.4, 165.3, 164.0, 162.6, 159.7, 159.3, 158.9, 158.5, 149.9, 149.4, 148.3, 147.9, 146.2, 145.5, 144.0, 143.6, 143.0, 138.9, 135.9, 132.0, 131.1, 130.7, 130.4, 130.1, 127.94, 127.91, 122.4, 120.4, 119.5, 117.6, 116.6, 114.8, 111.9, 91.2, 55.06, 54.8, 54.6, 54.4, 54.2, 52.3, 51.8, 51.5, 49.4, 49.2 (other peaks likely obscured by solvent resonances); HRMS (ESI-TOF) *m*/*z* calculated for [C_40_H_34_N_9_O_5_S_3_]^+^ 816.184, found 816.184.

#### 4.4.6. Oxazole Peptoid Trimer **4b**

The general procedure was followed, using **1b** and 3-(aminomethyl)pyridine. Following purification by RP-HPLC using 20–55% gradient of solvent B in solvent A, 56 mg (48%) **4b** were isolated as a white powder. ^1^H-NMR (400 MHz, CD_3_OD) δ 9.14–8.21 (m, 12H), 8.15–7.75 (m, 3H), 7.63–7.13 (m, 5H), 5.57–4.48 (m, 12H, overlapping with HOD peak); ^13^C-NMR (100 MHz, CD_3_OD) δ 174.7, 164.2, 163.9, 163.7, 162.8, 161.5, 159.3, 158.9, 147.8, 146.8, 146.4, 144.3, 138.7, 137.10, 135.8, 134.9, 134.5, 133.3, 132.0, 130.1, 129.9, 128.6, 127.9, 119.6, 117.6, 116.7, 114.8, 111.9, 51.8, 51.6, 51.5, 49.9, 49.7, 49.4, 49.2, 19.0, 48.1, 44.7, 30.9; HRMS (ESI-TOF) *m*/*z* calculated for [C_40_H_34_N_9_O_8_]^+^ 768.253, found 768.254.

#### 4.4.7. Thiazole Peptoid Trimer **5**

The general procedure was followed, using **1a** and 4-phenylbutylamine. Following purification by RP-HPLC using 70–100% gradient of solvent B in solvent A, 56 mg (40%) **5** were isolated as a white powder. ^1^H-NMR (400 MHz, CD_3_CN) δ 8.25–7.82 (m, 3H), 7.56–6.56 (m, 20H), 5.31–4.45 (m, 6H), 3.89–3003 (m, 6H), 2.74–2.15 (m, 6H), 1.81–0.94 (m, 12H); ^13^C-NMR (100 MHz, CD_3_CN) δ 172.6, 170.6, 169.3, 169.1, 168.3, 168.0, 167.7, 164.7, 163.9, 162.6, 150.3, 147.3, 147.0, 143.5, 143.4, 143.1, 137.2, 130.6, 130.5, 130.3, 129.6, 129.3, 129.4, 127.9, 127.5, 126.8, 126.7, 51.5, 51.4, 51.0, 50.7, 49.2, 49.0, 48.7, 47.8, 36.1, 35.8, 30.4, 29.7, 29.5, 29.3, 29.0, 27.6; HRMS (ESI-TOF) *m*/*z* calculated for [C_52_H_55_N_6_O_5_S_3_]^+^ 938.332, found 938.332.

#### 4.4.8. Thiazole Peptoid Trimer **6**

The general procedure was followed, using **1a** and isopropylamine. Following purification by RP-HPLC using 50–80% gradient of solvent B in solvent A, 15 mg (15%) **6** were isolated as a white powder. ^1^H-NMR (400 MHz, CD_3_CN) δ 8.27–8.11 (br s, 1 H), 8.03–7.74 (m, 2H), 7.61–7.23 (m, 5H), 6.96–5.81 (br s, 5H), 5.41–4.40 (m, 8H), 4.20–3.83 (br s, 1H), 1.49–0.72 (m, 18H); ^13^C NMR (100 MHz, CD_3_CN) δ 172.7, 171.2, 169.9, 169.7, 165.8, 162.4, 159.7, 159.3, 150.3, 149.9, 146.4, 137.6, 130.6, 130.3, 129.7, 127.2, 125.4, 125.1, 120.8, 115.1, 52.0, 51.4, 48.7, 47.3, 43.9, 43.7, 43.2, 30.4, 21.6, 21.3, 20.3; HRMS (ESI-TOF) *m*/*z* calculated for [C_31_H_37_N_6_O_5_S_3_ + H]^+^ 668.191, found 668.192.

#### 4.4.9. Thiazole Peptoid Trimer **7**

The general procedure was followed, using **1a** and 4-(2-aminoethyl)morpholine and *tert*-butyl *N*-(2-aminoethyl)carbamate with the following exception. Following *N*-benzoylation, a solution of 95% TFA, 2.5% water, and 2.5% triisopropylsilane was added to the resin, and the mixture was gently agitated, then allowed to sit for 1 h at room temperature. The solution and CH_2_Cl_2_ washes were then combined, concentrated by rotary evaporation, then dissolved in acetonitrile and water, frozen, and lyophilized. Following purification by RP-HPLC using 20–40% gradient of solvent B in solvent A, 59 mg (63%) **7** were isolated as a white powder. ^1^H-NMR (400 MHz, CD_3_CN) δ 11.92–9.55 (br s, 3H), 8.48–7.73 (m, 6H), 7.61–7.16 (m, 5H), 5.61–4.56 (m, 6H), 4.38–2.68 (m, 28H); ^13^C-NMR (100 MHz, CD_3_CN) δ 173.8, 173.0, 170.5, 168.0, 165.5, 165.4, 164.5, 162.1, 161.8, 161.4, 161.1, 149.8, 149.3, 148.8, 136.0, 131.4, 129.6, 128.8, 127.9, 122.0, 119.2, 116.2, 113.3, 64.7, 64.5, 55.2, 54.9, 53.2, 51.9, 51.5, 48.6, 44.7, 42.9, 42.5, 41.4, 39.4, 38.6, 30.4; HRMS (ESI-TOF) *m*/*z* calculated for [C_36_H_46_N_9_O_7_S_3_]^+^ 812.268, found 812.268.

#### 4.4.10. Thiazole Peptoid Trimer **8**

The general procedure was followed, using **1a** and propargylamine, 2-methoxyethylamine, and 4-(2-aminoethyl)morpholine. Following purification by RP-HPLC using 30–90% gradient of solvent B in solvent A, 44 mg (39%) **8** were isolated as a white powder. ^1^H-NMR (400 MHz, CD_3_CN) δ 8.29–8.12 (m, 1H), 8.11–7.92 (m, 2H), 7.55–7.18 (m, 5H), 5.49–4.69 (m, 7H), 4.03–2.89 (m, 23H); ^13^C-NMR (100 MHz, CD_3_CN) δ 173.6, 170.0, 168.9, 165.8, 164.8, 163.7, 162.1, 161.8, 150.7, 147.9, 137.6, 131.2, 130.7, 130.0, 128.5, 127.9, 127.7, 73.0, 71.7, 71.5, 59.6, 53.1, 52.7, 50.9, 50.6, 50.4, 48.1; HRMS (ESI-TOF) *m*/*z* calculated for [C_34_H_38_N_7_O_7_S_3_]^+^ 751.192, found 751.192.

#### 4.4.11. Thiazole Peptoid Trimer **9**

The general procedure was followed with modifications as noted below using **1a** and 2-methoxyethylamine, and 2-(2,2′:6′,2′′-Terpyridine-4′-yloxyl)ethylamine. Following benzoylation of the *N*-terminus and subsequent washing steps, 2 mL of a 20% solution of 4-methylpiperidine in DMF was added to reverse undesired benzoylation of the terpyridine side chain. The reaction vessel was shaken for 20 min, then the resin was washed with DMF before cleaving **9** from the resin as described in the general procedure. Following purification by RP-HPLC using 50–80% gradient of solvent B in solvent A, 83 mg (30%) **9** were isolated as a white powder. ^1^H-NMR (400 MHz, CD_3_CN) δ 11.40 (br s, 1H), 9.09, (d, 2H), 8.80–8.40 (m, 4H), 8.24–7.68 (m, 7H), 7.61–7.32 (m, 5H), 5.48–3.05 (m, 24H); ^13^C-NMR (100 MHz, CD_3_CN) δ 171.7, 167.9, 166.3, 163.7, 162.5, 161.5, 160.1, 159.8, 159.4, 159.1, 149.3, 148.5, 147.3, 145.9, 144.7, 144.4, 135.3, 129.5, 128.6, 128.3, 127.3, 126.6, 126.0,123.9, 120.4, 117.5, 114.6, 111.7, 110.3, 70.9, 69.7, 66.8, 57.7, 51.0, 50.8, 48.4, 47.8, 46.2; HRMS (ESI-TOF) *m*/*z* calculated for [C_45_H_44_N_9_O_8_S_3_]^+^ 934.247, found 934.246.

### 4.5. Evaluation of Zn^2+^ Binding by **9**

#### 4.5.1. Preparation of a Stock Solution of **9**

A precisely-weighed quantity of purified, lyophilized thiazole peptoid **9** was dissolved in 1:1 dioxane:water at an approximate concentration of 5 mM. Additional small volumes of 10 mM Tris buffer, pH 7.5 were added gradually to effect complete dissolution of **9**, and the final concentration was determined from the mass of **9** and the total volume of solution.

#### 4.5.2. Zn^2+^ Titrations to **9** Monitored by UV-Vis Spectroscopy

Solutions of 50 μM **9** (diluted from the stock) and 0–150 μM and ZnCl_2_ (diluted from a 5 mM stock solution in 10 mM Tris buffer, pH 7.5) were prepared in 10 mM Tris buffer, pH 7.5 to a final volume of 500 μL. Each solution was mixed well, then 150 μL was distributed to each of three wells in a transparent flat bottom, low volume, acrylic 96-well plate. The UV-Visible spectrum was recorded for each well of the plate by a Molecular Devices SpectraMax i3x plate reader. Spectra were collected from 230 nm to 900 nm every 1 nm. Spectra were averaged for the three wells containing identical solutions.

## Figures and Tables

**Table 1:** Yields and purities of trimeric heteroarylopeptoids prepared according to Scheme 1.

Compound	Structure	Crude Yield	Crude Purity ^1^	Isolated Yield ^4^
**2a** (X = S)	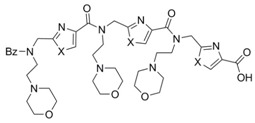	**2a**: 79%	**2a**: 85%	**2a**: 61%
**2b** (X = O)		**2b**: 60%	**2b**: 60%	**2b**: 33%
**3a** (X = S)	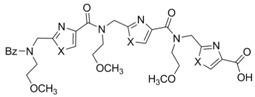	**3a**: 58%	**3a**: 81%	**3a**: 13%
**3b** (X = O)		**3b**: 79%	**3b**: 77%	**3b**: 31%
**4a** (X = S)	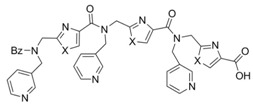	**4a**: 80%	**4a**: 77%	**4a**: 51%
**4b** (X = O)		**4b**: 63%	**4b**: 84%	**4b**: 48%
**5**	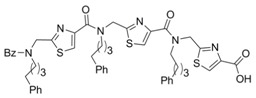	65%	89%	40%
**6**	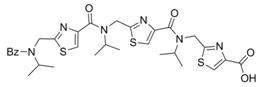	52%	80%	15%
**7**	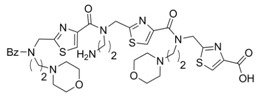	110% ^2^	98%	63%
**8**	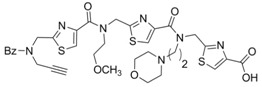	ND ^3^	ND ^3^	39%
**9**	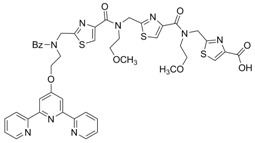	67%	ND ^3^	29%
**10**	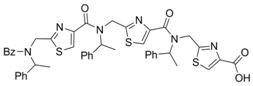	-- ^5^	--	--

^1^ Determined by integration of crude HPLC chromatogram; ^2^ Cleavage from resin and amine deprotection using 95% TFA, 2.5% triisopropylsilane, 2.5% water. Isolated yield >100% is likely due to the presence of ammonium trifluoroacetate salt; ^3^ ND = not determined; ^4^ Isolated yields were calculated following RP-HPLC purification; ^5^ Yield was negligible using these conditions owing to incomplete acylation reactions.

## Supplementary Materials

The following are available online. Scheme S1: Synthesis of functionalized azole building blocks **1a** and **1b**. Figure S1: Crude analytical HPLC chromatograms, Figure S2: Analytical HPLC chromatograms of purified compounds, Figure S3: ^1^H and ^13^C-NMR spectra of **2**–**9**, Figure S4: UV spectra of **3a** in the presence of increasing [Zn^2+^], Figure S5: Variable temperature ^1^H-NMR spectra of **6**.
