# Supplementary material for: Solid-Phase Synthesis of Azole-Comprising Peptidomimetics and Coordination of a Designed Analog to Zn2+
Source: Molecules. 2018 Apr 28;23(5):1035. doi: 10.3390/molecules23051035 (PMC6102547; doi:10.3390/molecules23051035)
Supplement: Supplementary file 1 [file molecules-23-01035-s001.pdf]

Supporting Information for:

Solid-phase synthesis of azole-comprising peptidomimetics and coordination of a designed analog to Zn^{2+}

Aanchal Mohan, Allyson H. M. Koh, Gregory Gate, Anna L. Calkins, Kyra N. McComas, and Amelia A. Fuller

Department of Chemistry & Biochemistry, Santa Clara University, 500 El Camino Real, Santa Clara, CA 95053, United States

Table of Contents:

**Scheme S1.** Synthesis of functionalized azole building blocks **1a** and **1b**	S1
**Figure S1.** Crude analytical HPLC chromatograms	S2-S6
**Figure S2.** Analytical HPLC chromatograms of purified compounds	S7-S12
**Figure S3.** ^{1}H and ^{13}C NMR spectra of **2-9**	S13-S34
**Figure S4.** UV spectra of **3a** in the presence of increasing [Zn^{2+}]	S35
**Figure S5.** Variable temperature ^{1}H NMR spectra of **6**	S36

Scheme S1. Synthesis of functionalized azole building blocks **1a** and **1b**. Procedures followed those reported in: Aditya, A.; Kodadek, T. *ACS Comb. Sci.* **2012**, *14*, 164–169.

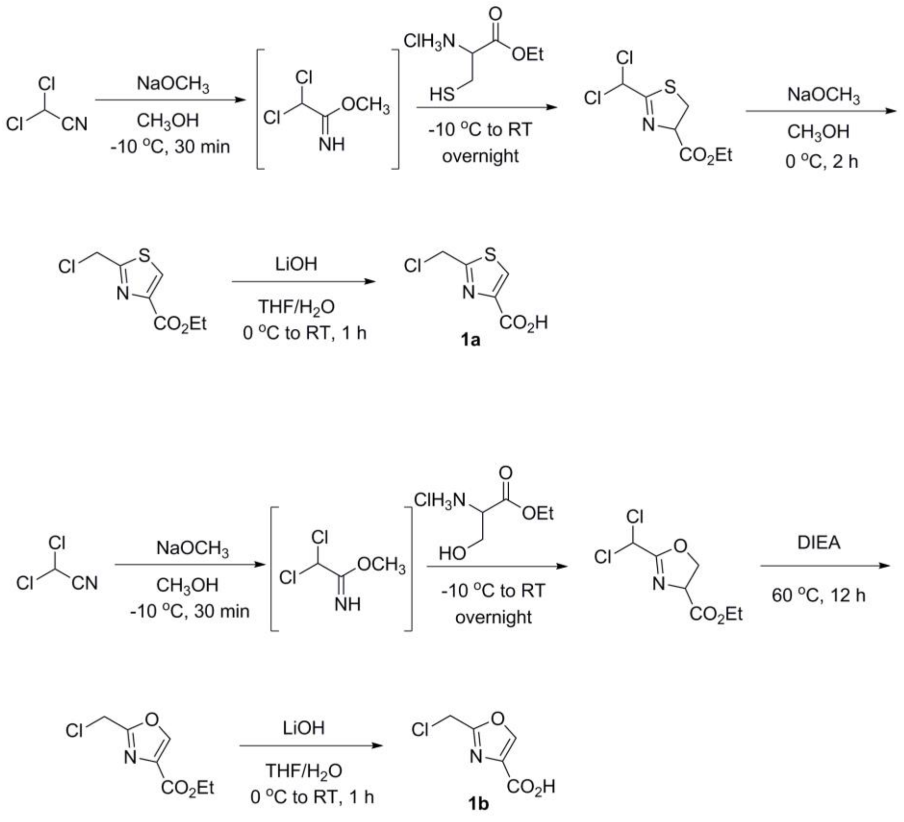

Figure S1. Crude analytical HPLC chromatograms

All Peaks were detected at 220 nm by RP-HPLC using 5-90% gradient of solvent B to solvent A.

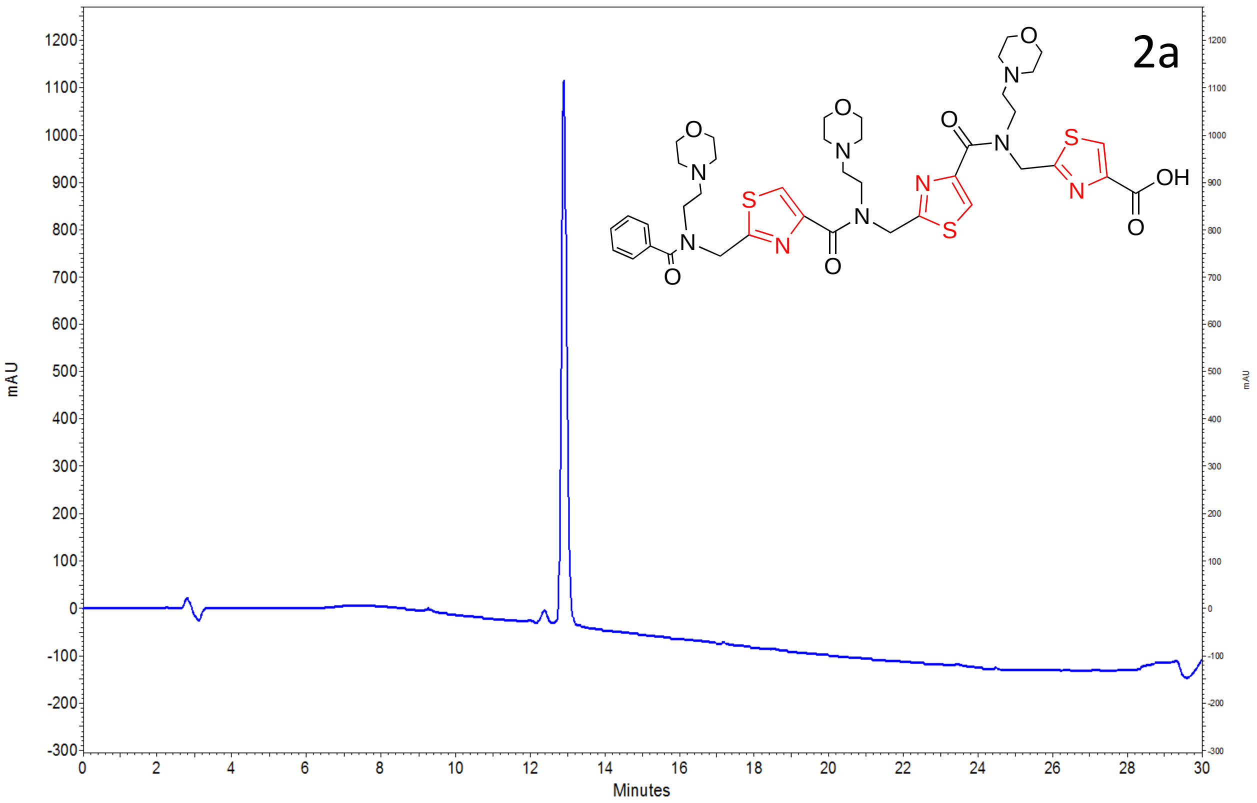

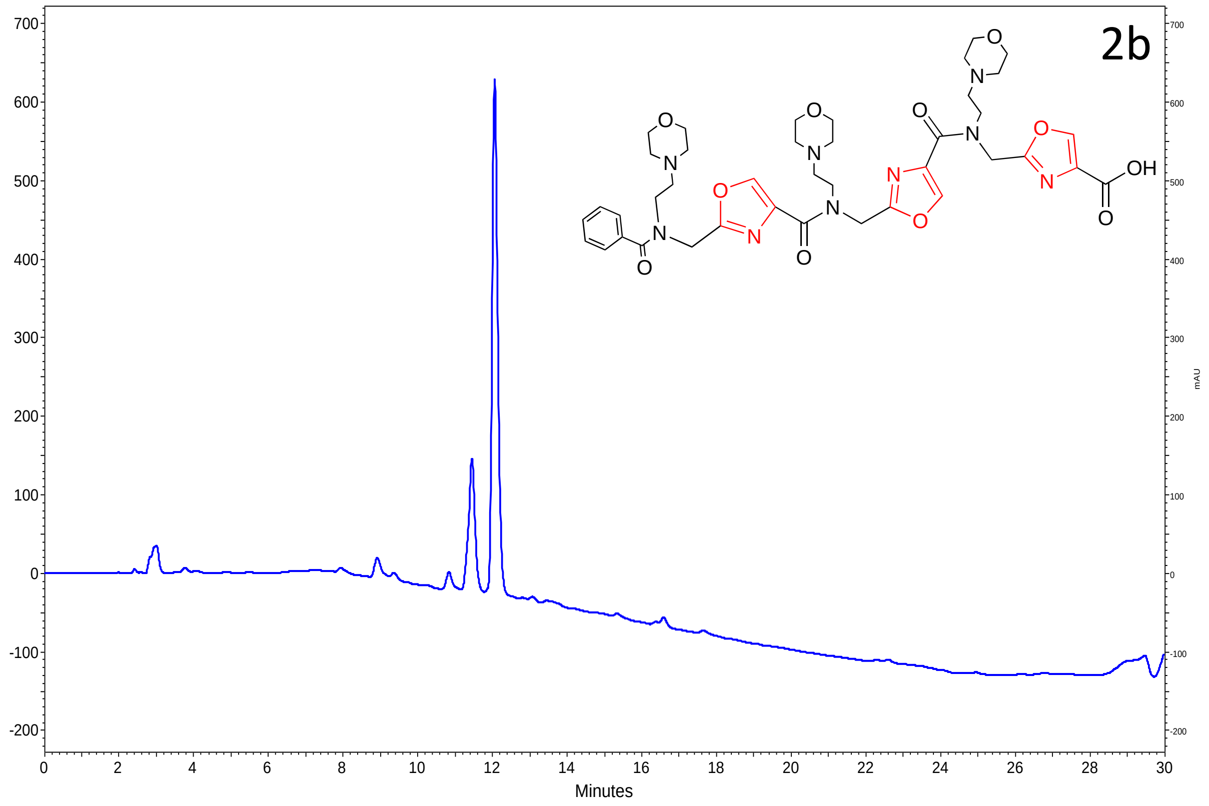
2b
Minutes
mAU
OH

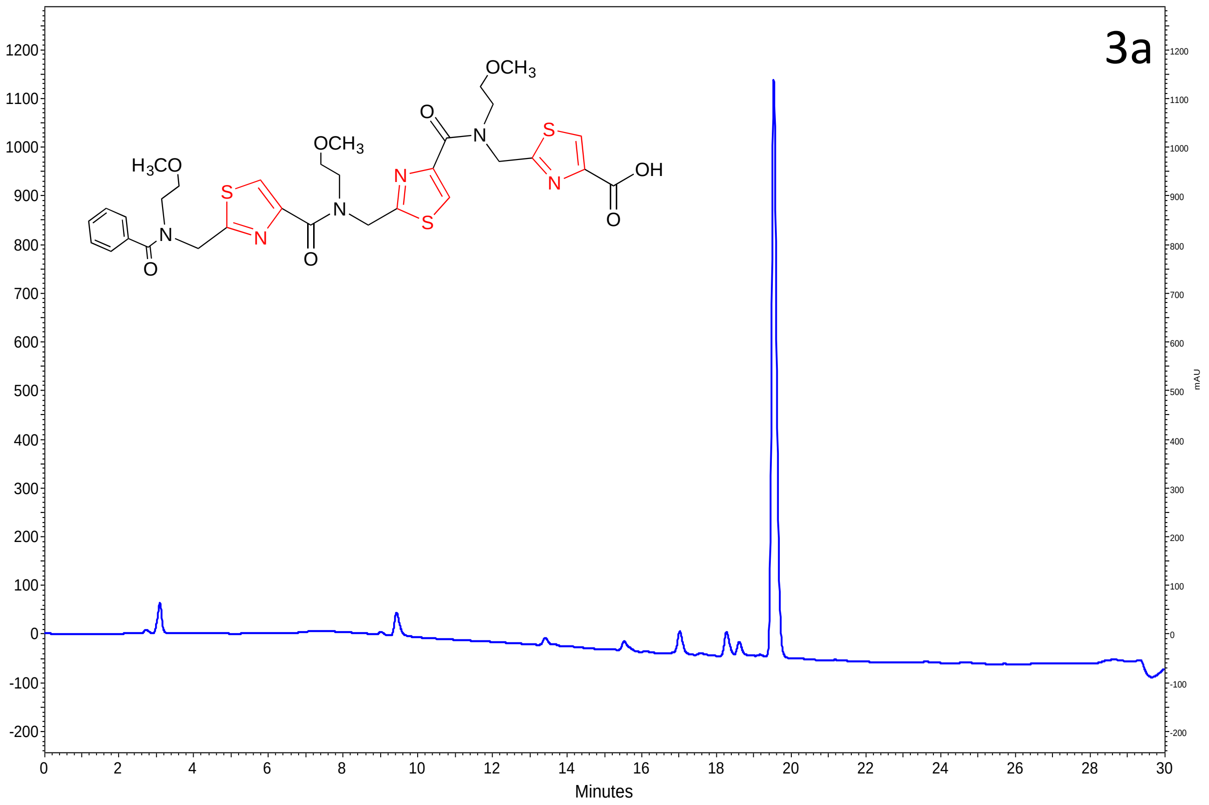
3a
Minutes
mAU
OCH3
H3CO
OH

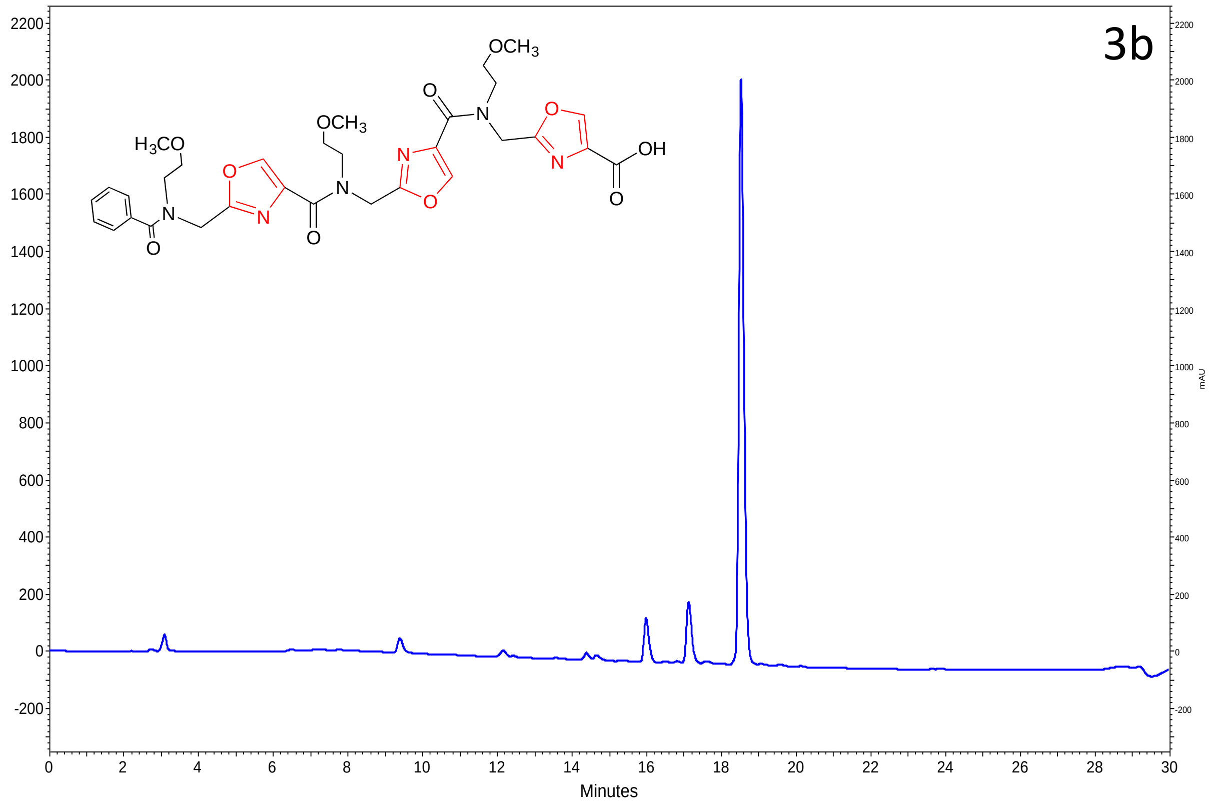

3b
Minutes
mAU

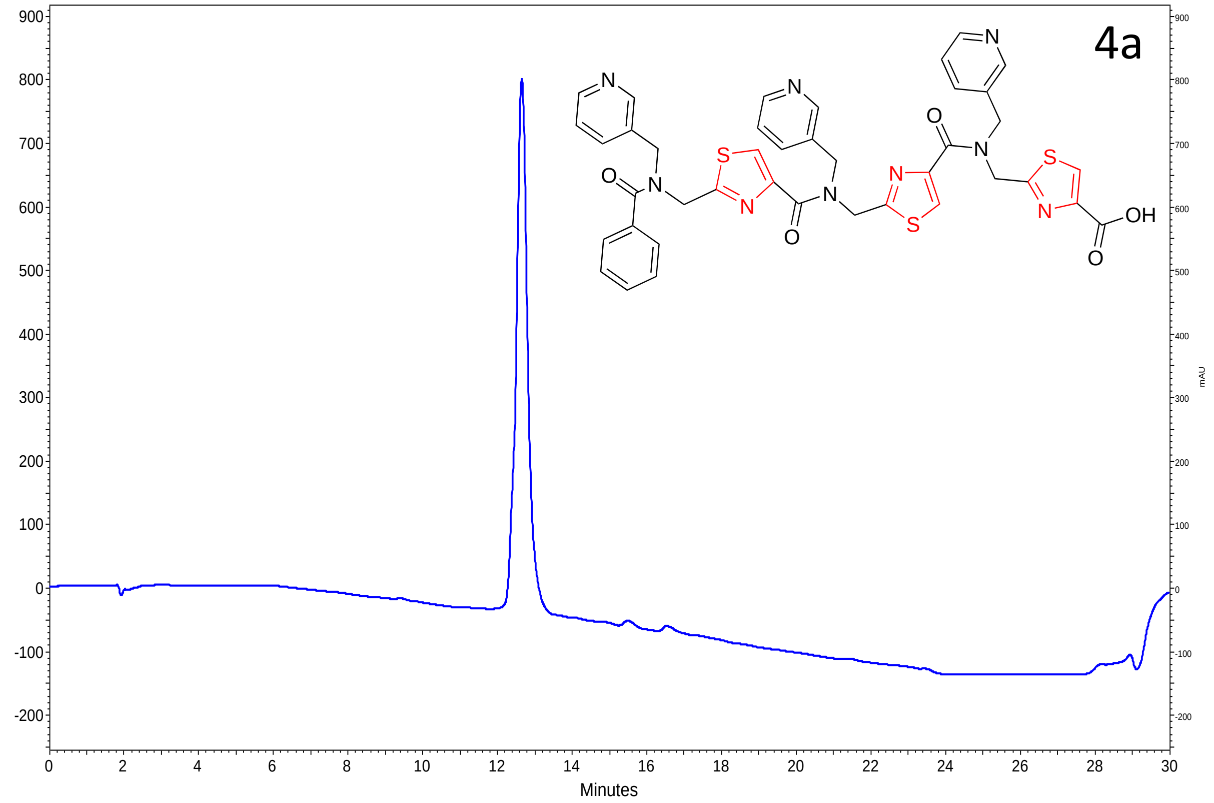

4a
Minutes
mAU

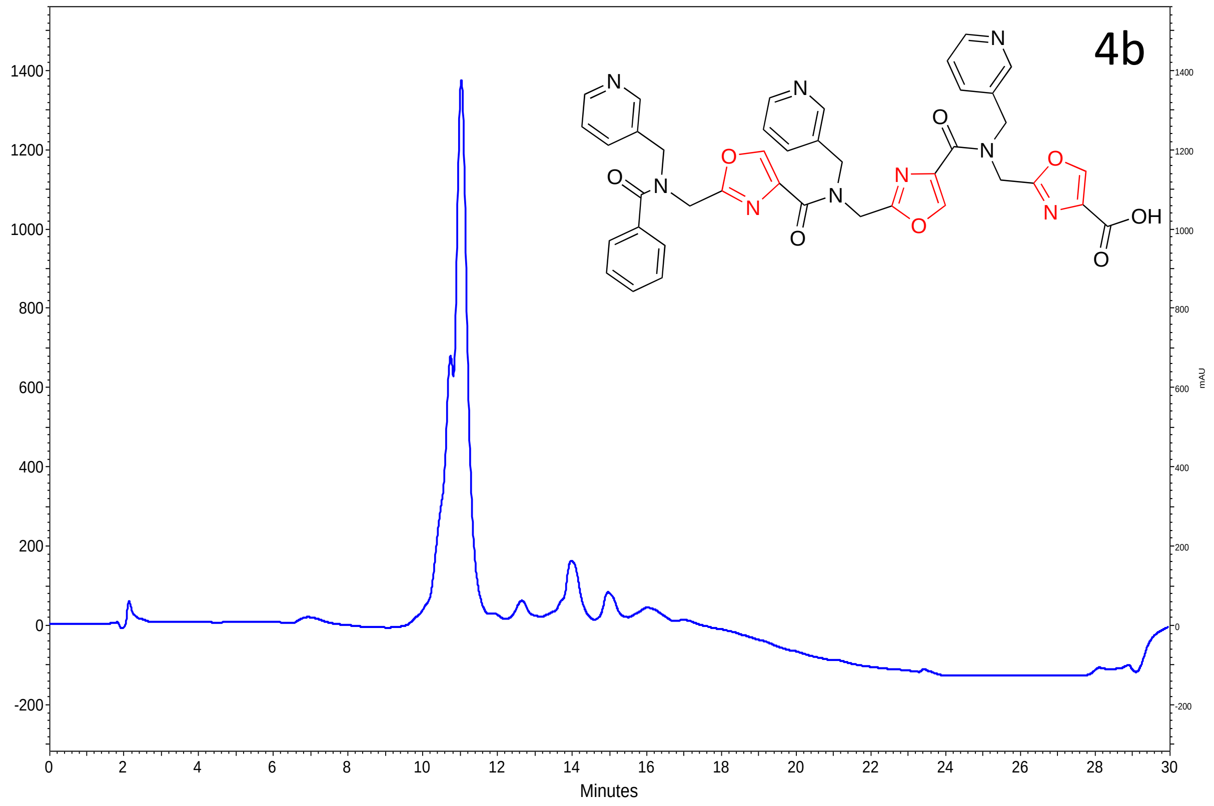

4b
Minutes

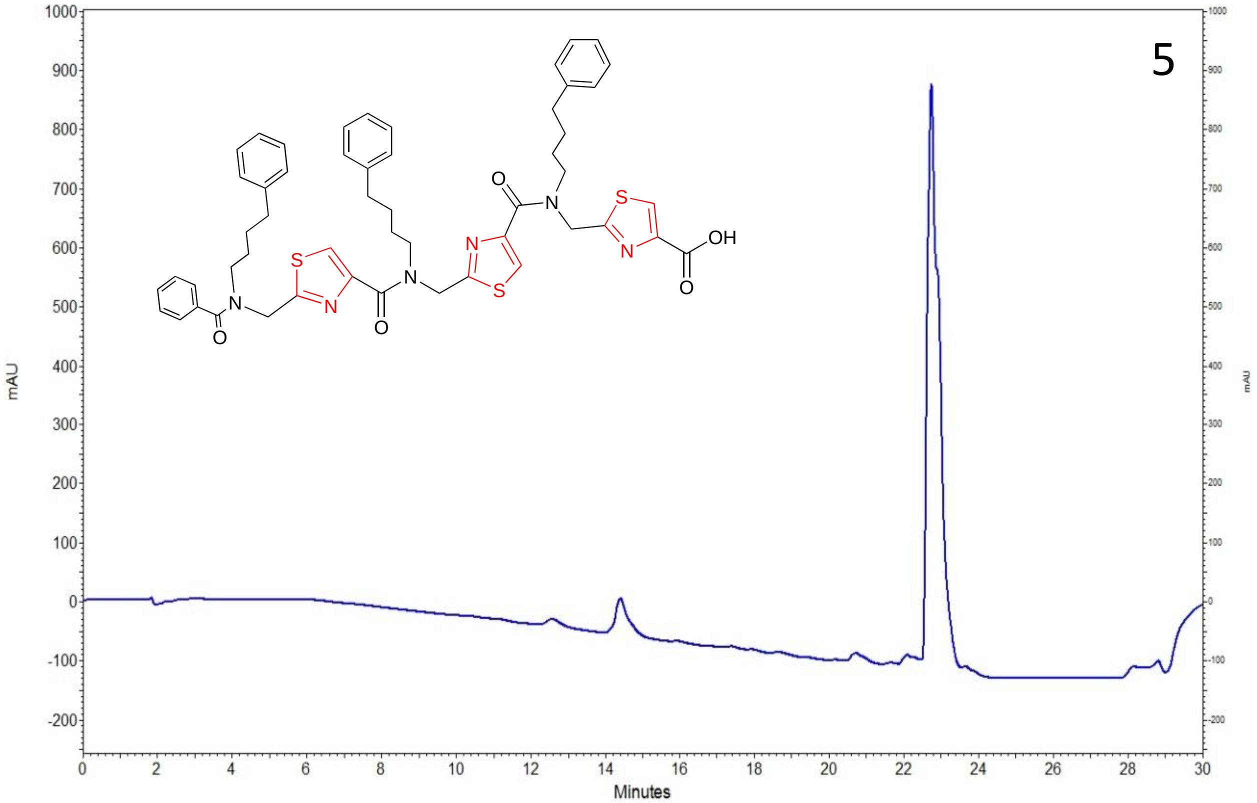

5
mAU
Minutes

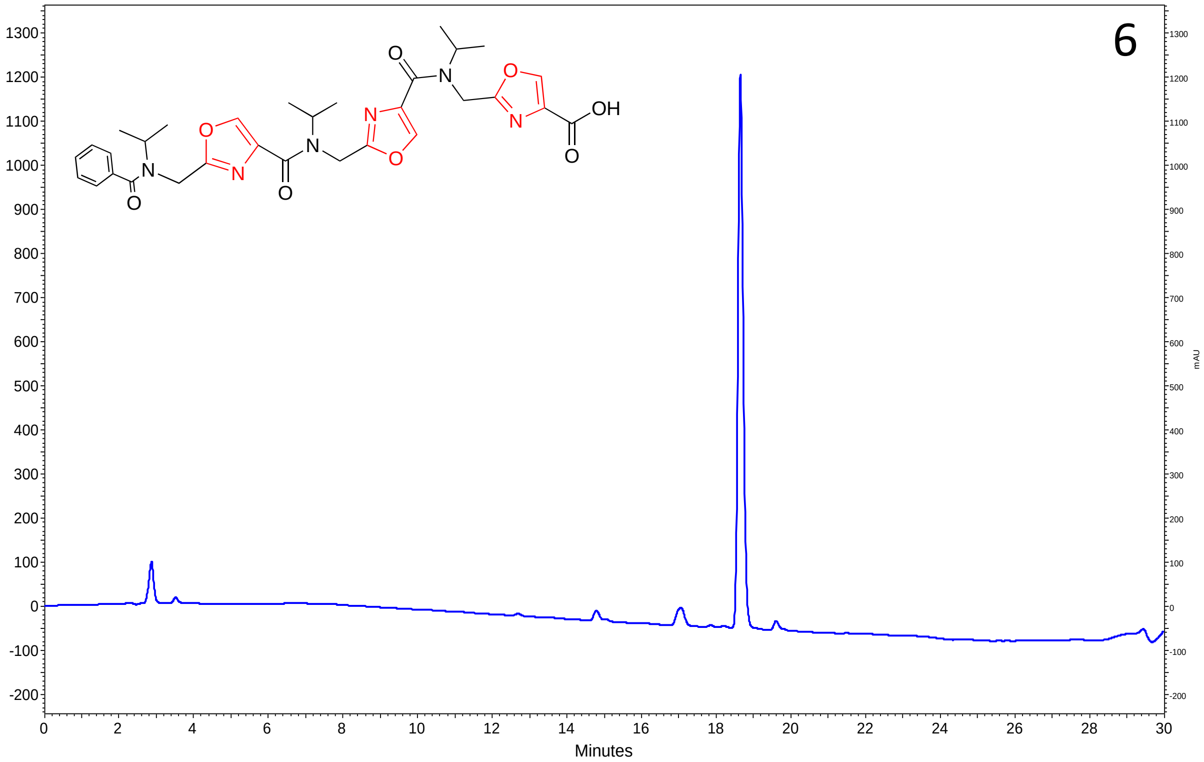

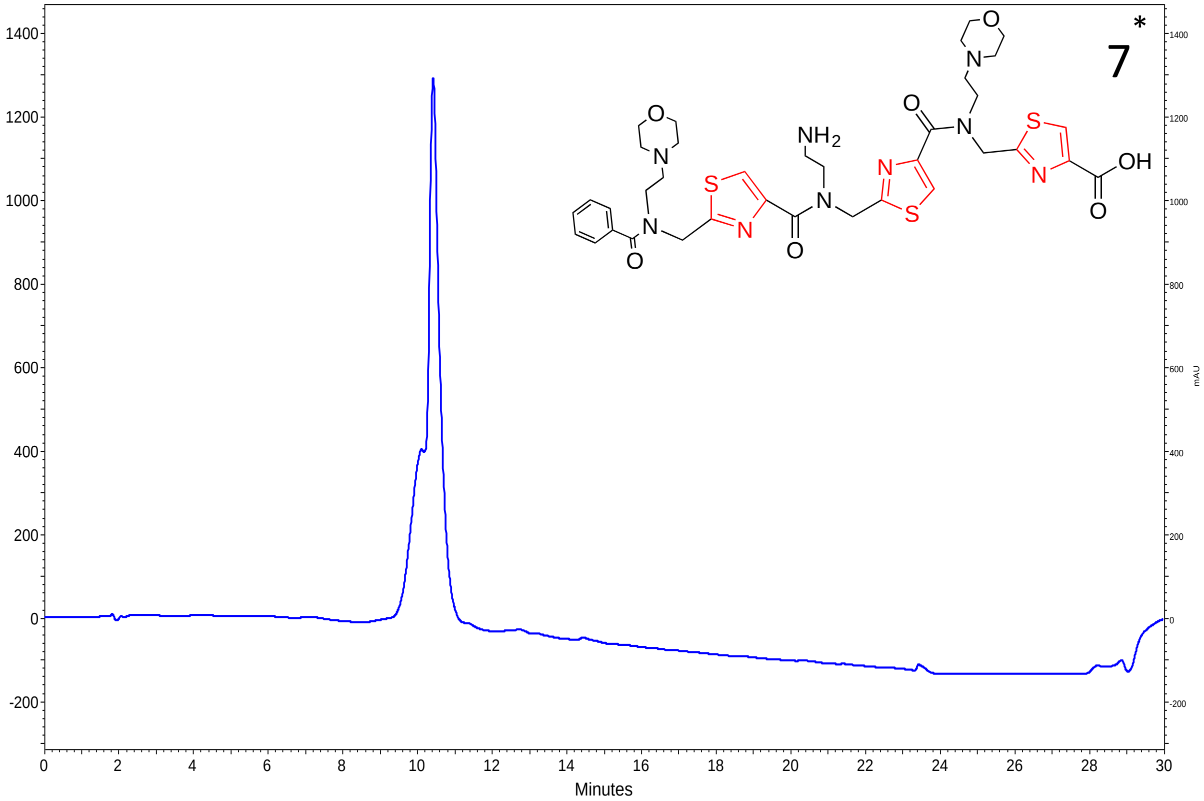

*The shoulder on the main peak was confirmed to be a partial separation of rotamers. The left and right sides of the peak were isolated in individual fractions. Re-injection of either fraction gave the same chromatogram seen above.

Analytical HPLC chromatograms of purified compounds

All Peaks were detected at 220 nm by RP-HPLC using 5-90% gradient of solvent B to solvent A.

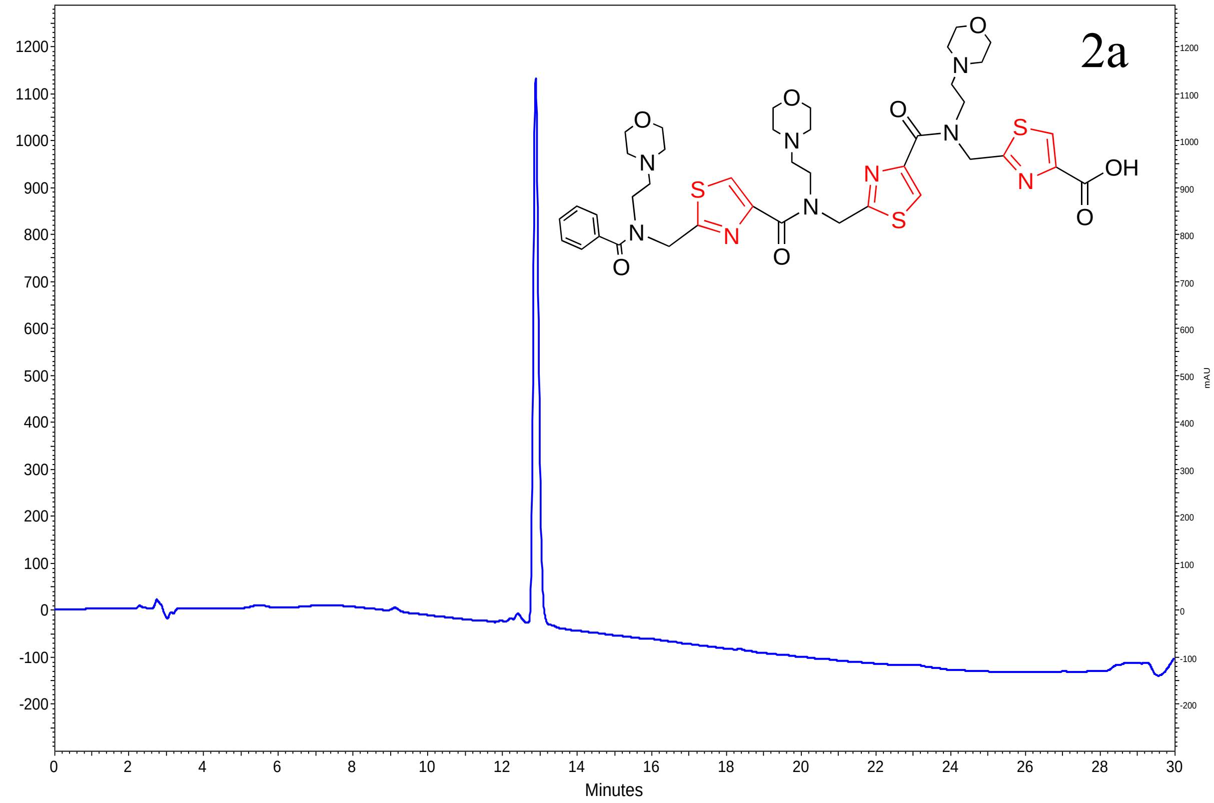

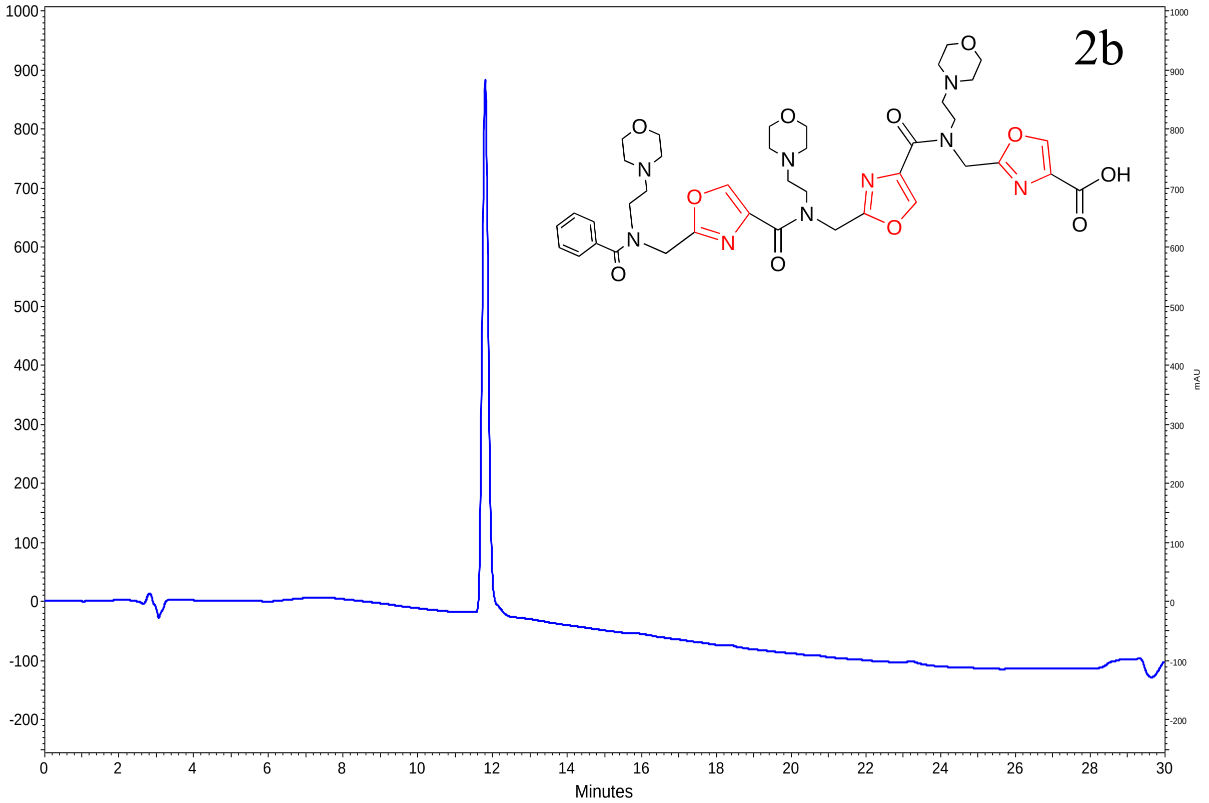

2b
mAU
Minutes

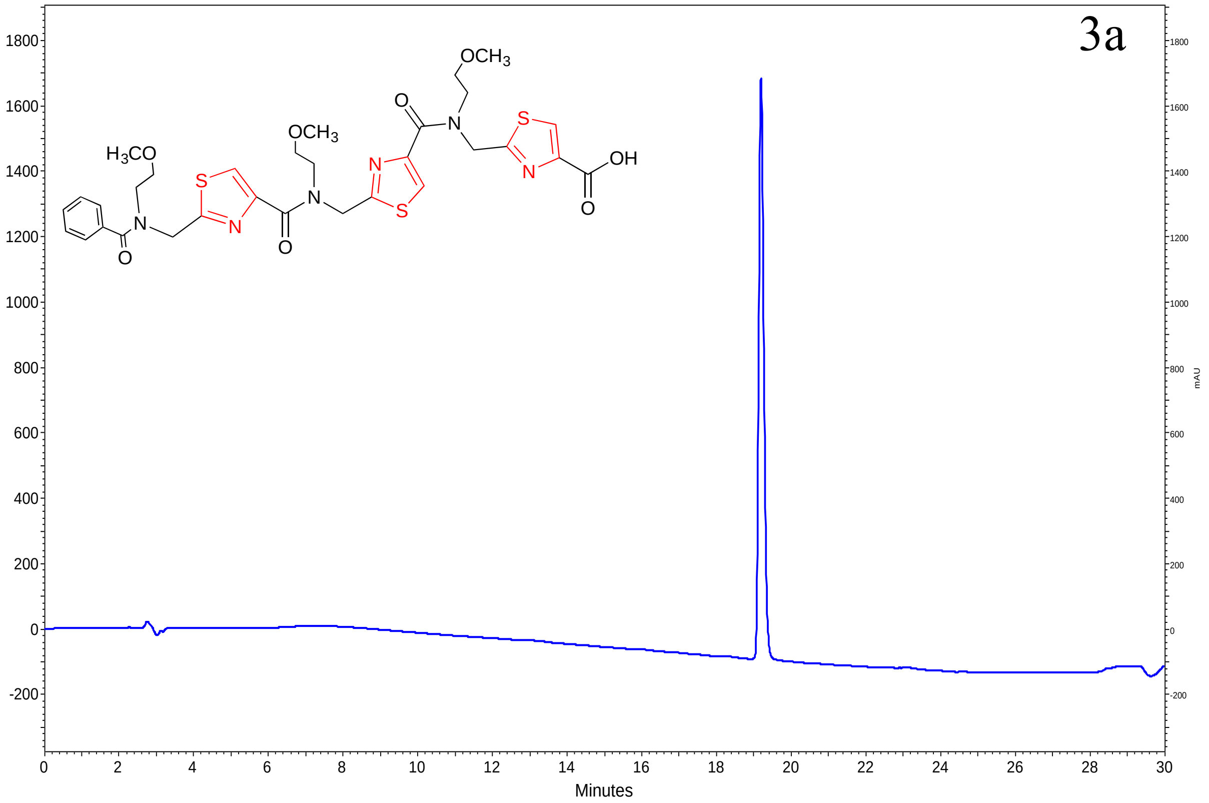

3a
mAU
Minutes

3b

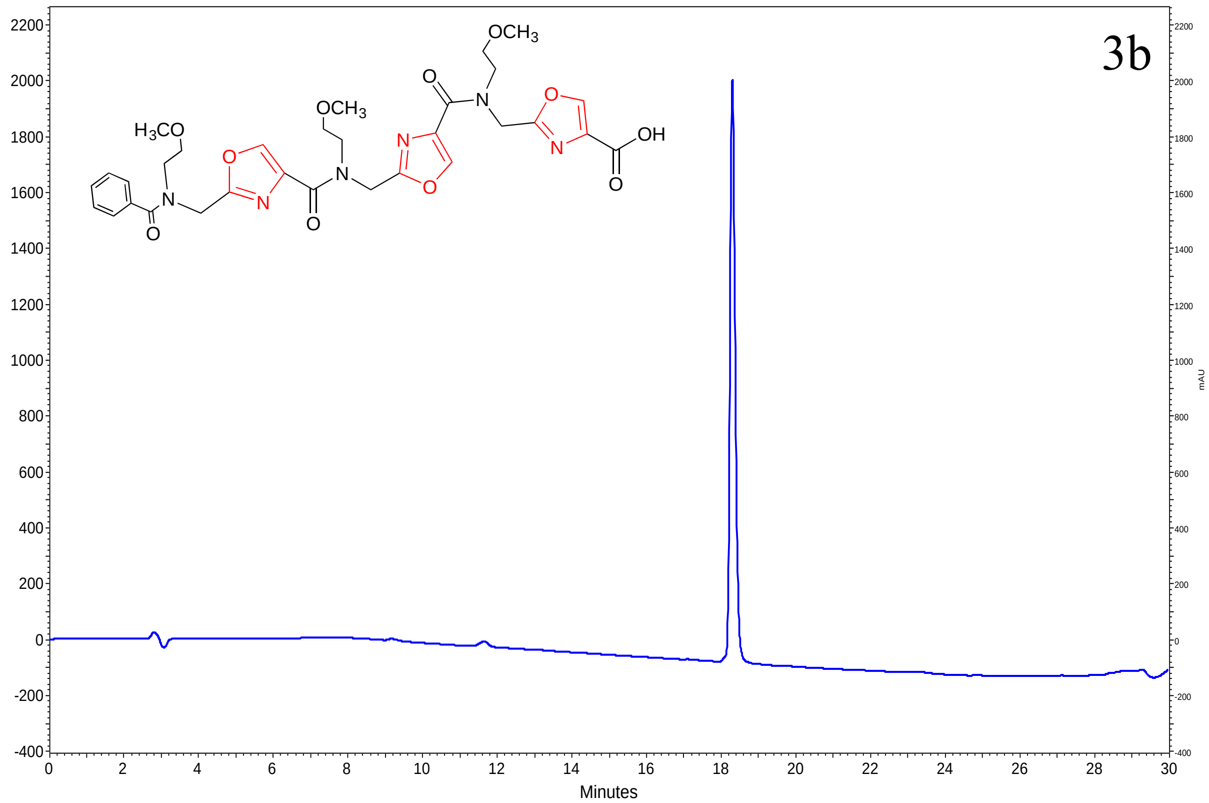

4a

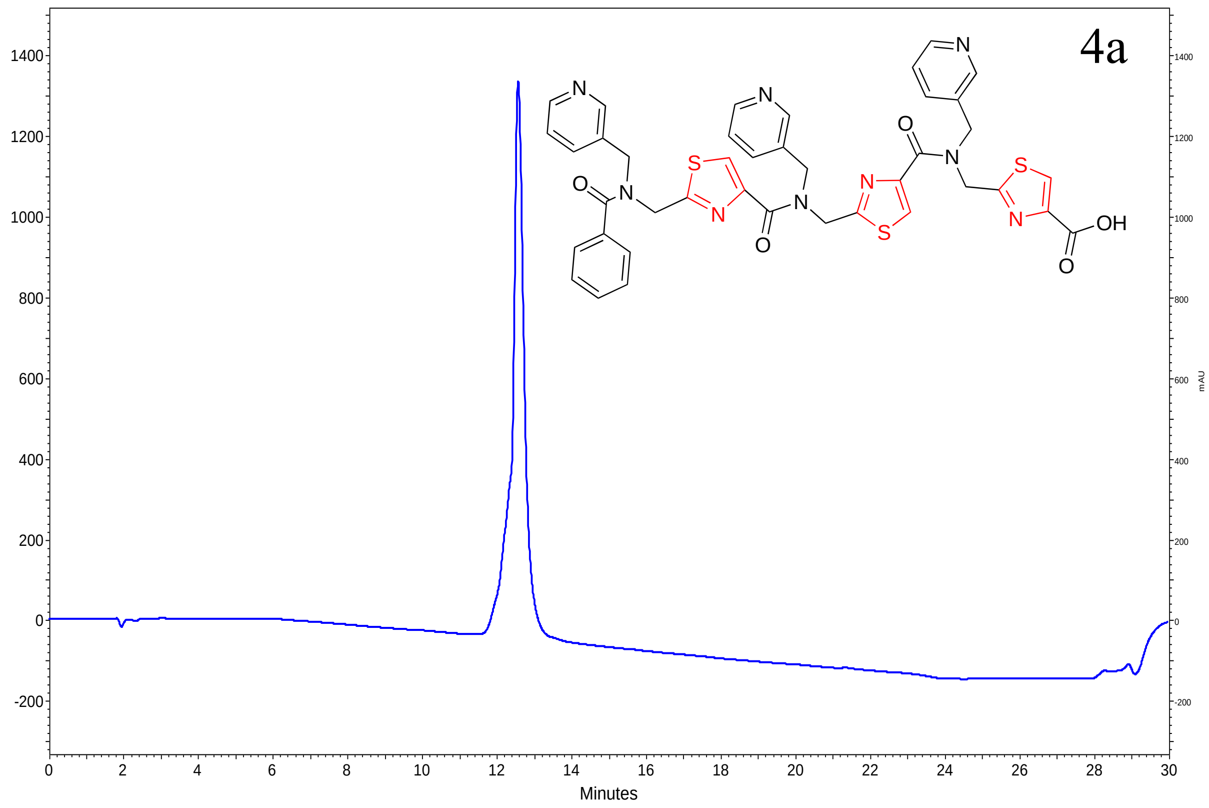

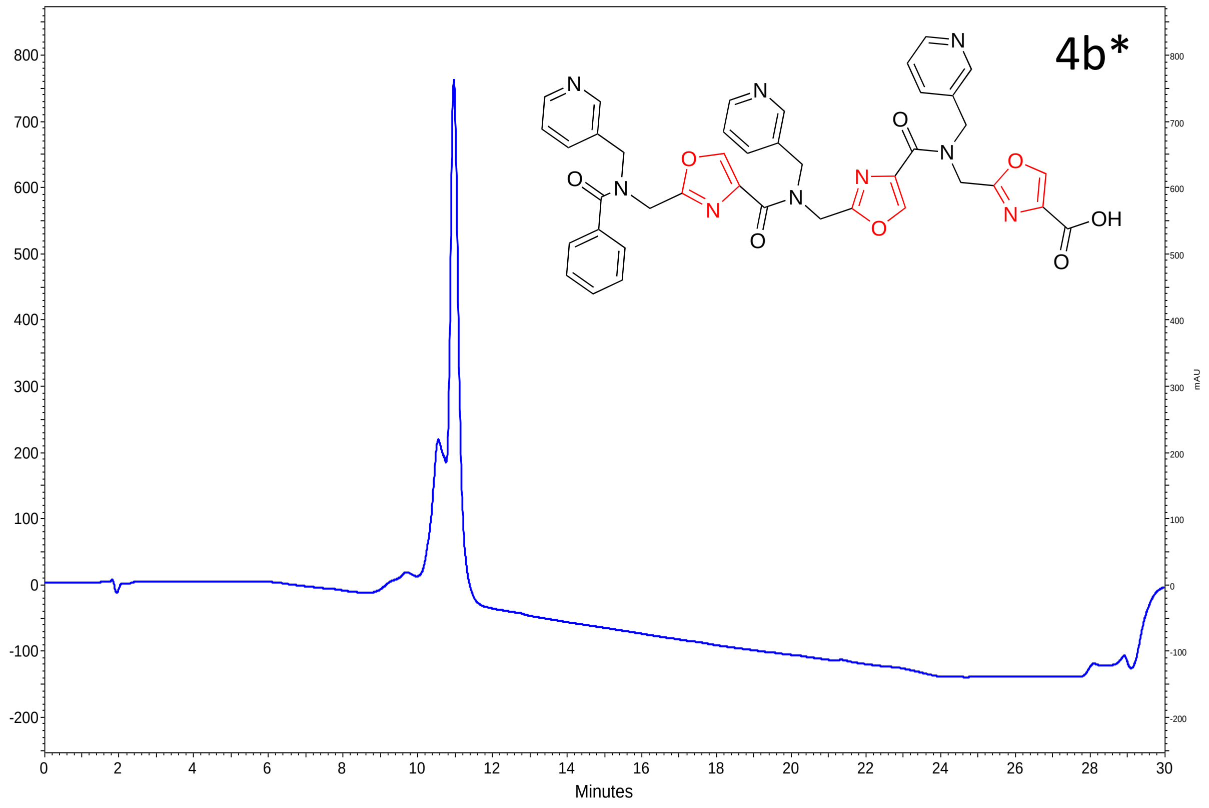
4b*
Minutes
mAU

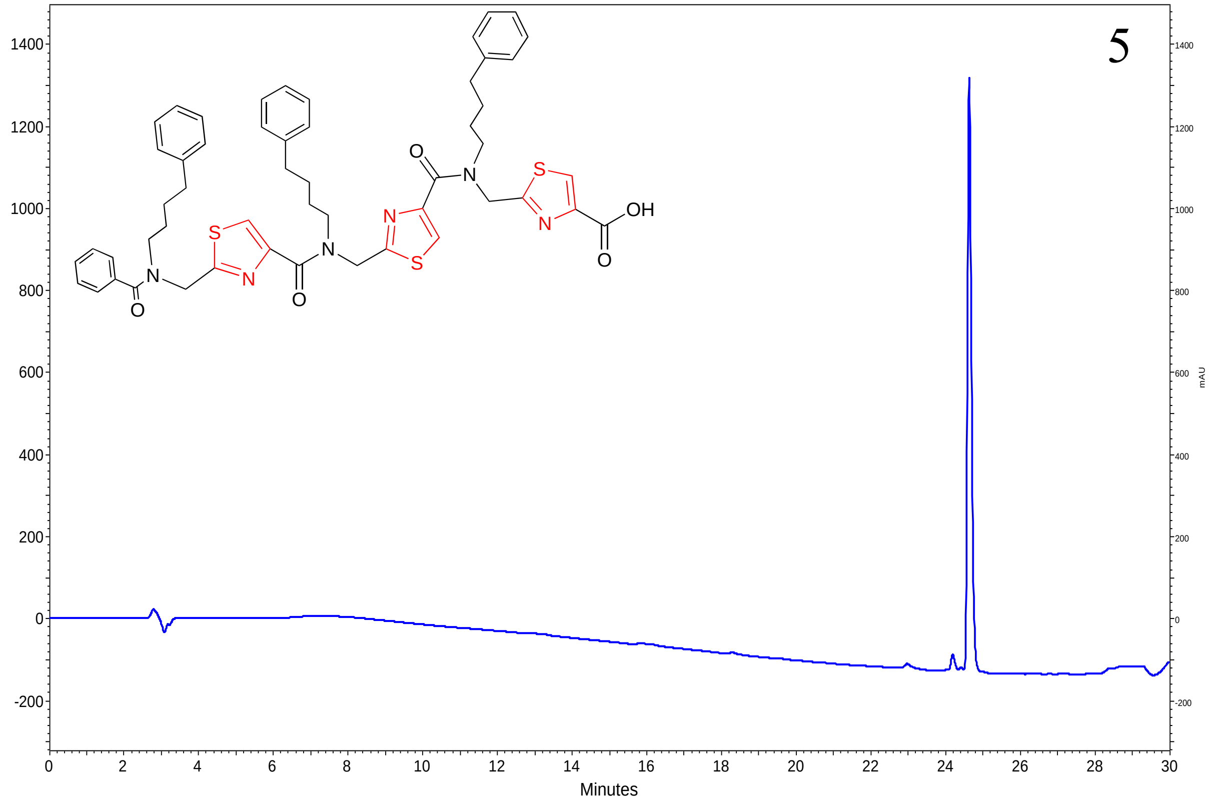
5
Minutes
mAU

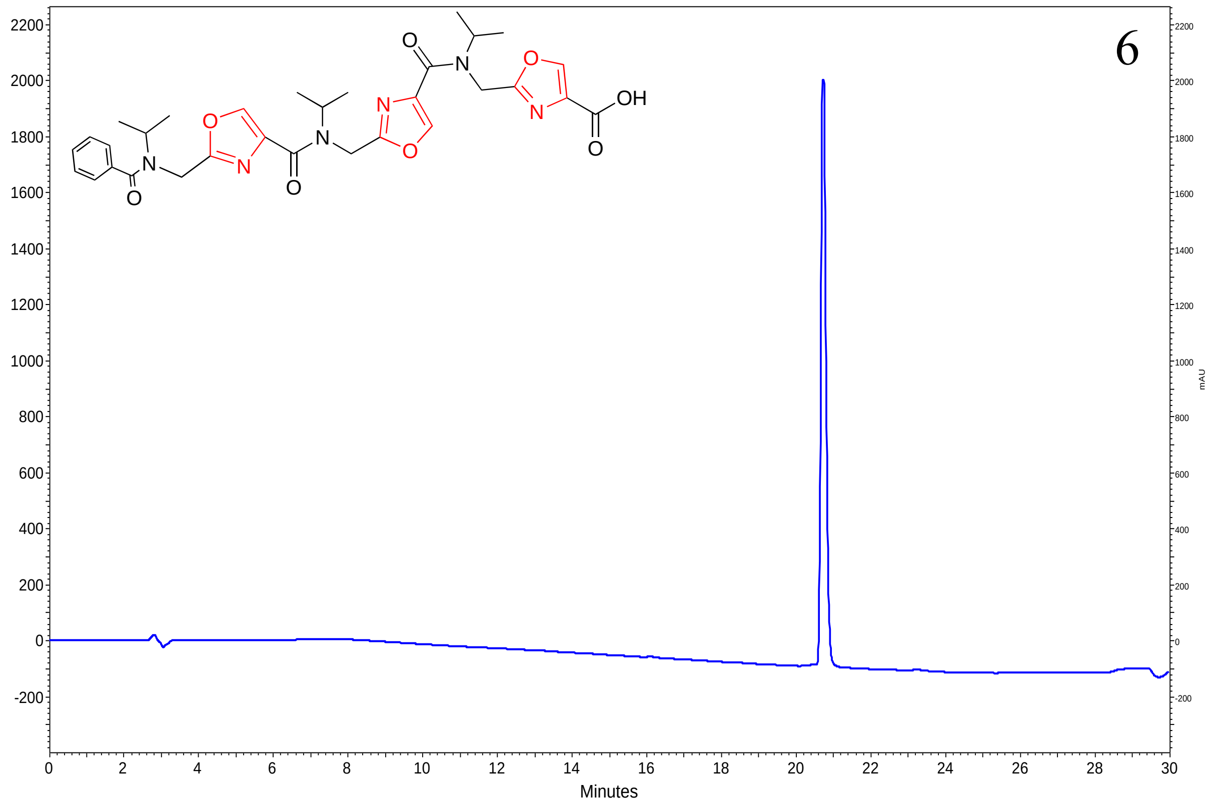
6
Minutes
mAU

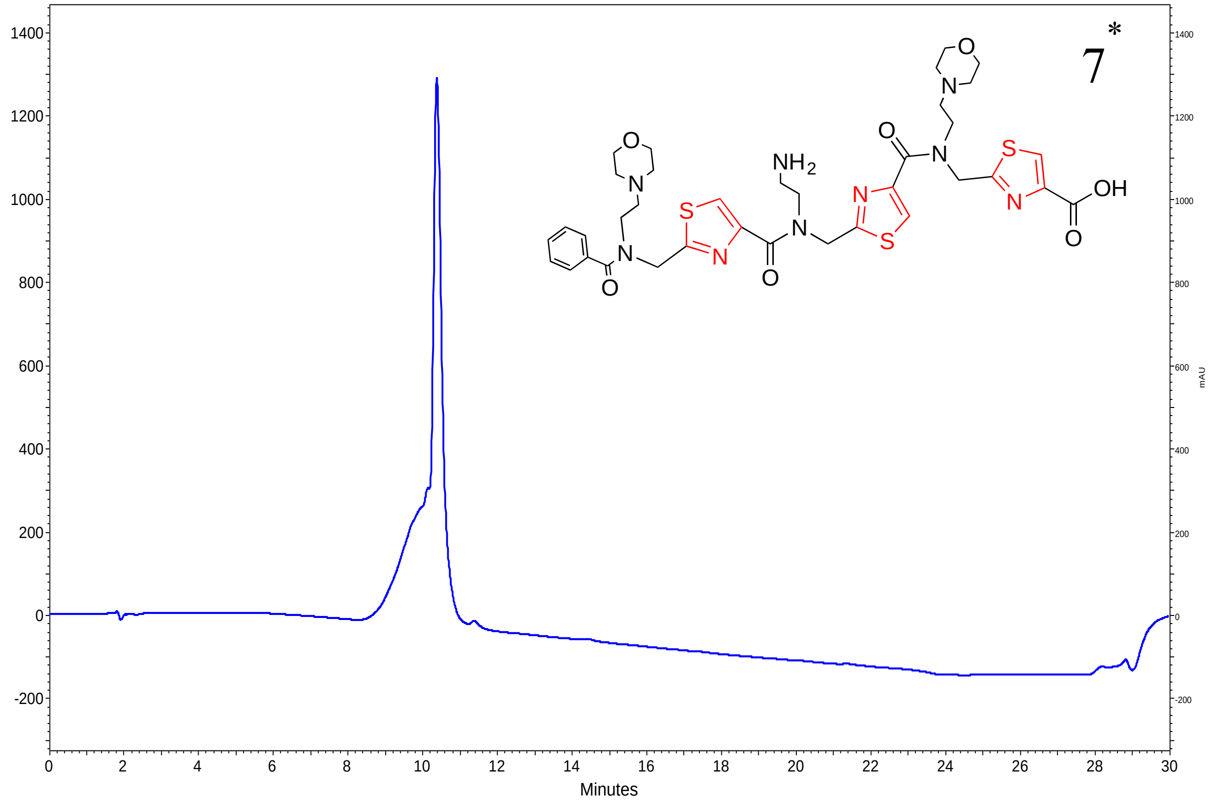
7*
Minutes
mAU

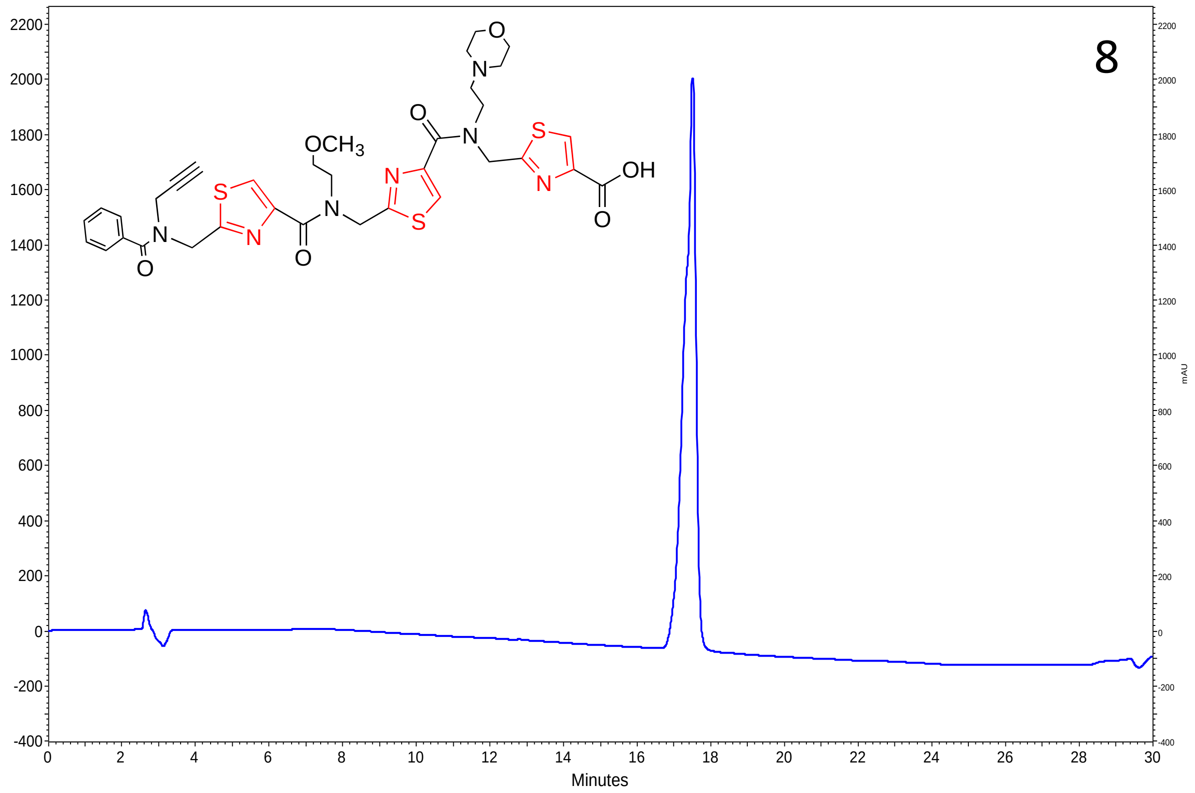

*The shoulder on the main peak was confirmed to be a partial separation of rotamers. The left and right sides of the peak were isolated in individual fractions. Re-injection of either fraction gave the same chromatogram seen above.

Figure S3. ^{1}H and ^{13}C NMR spectra

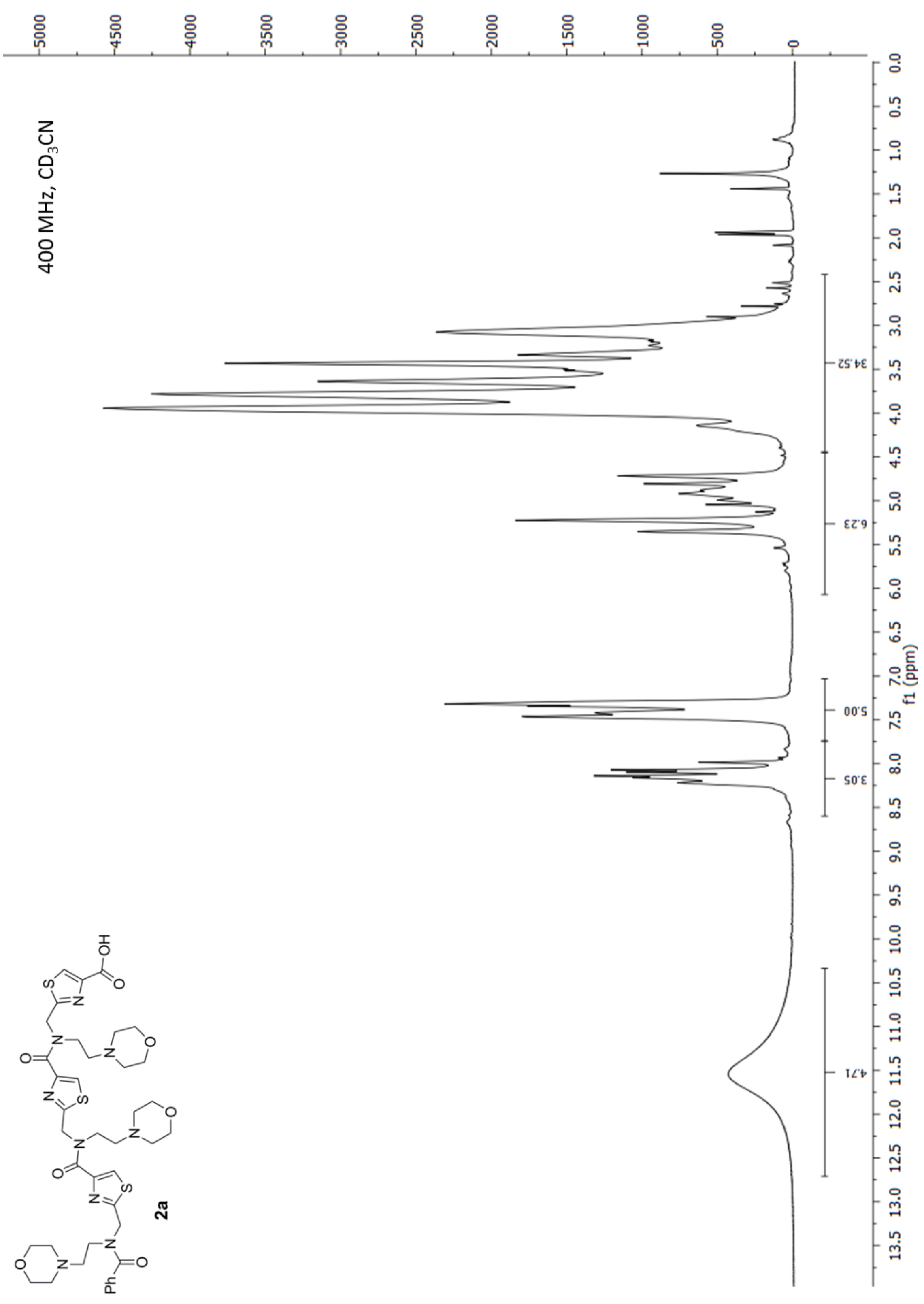

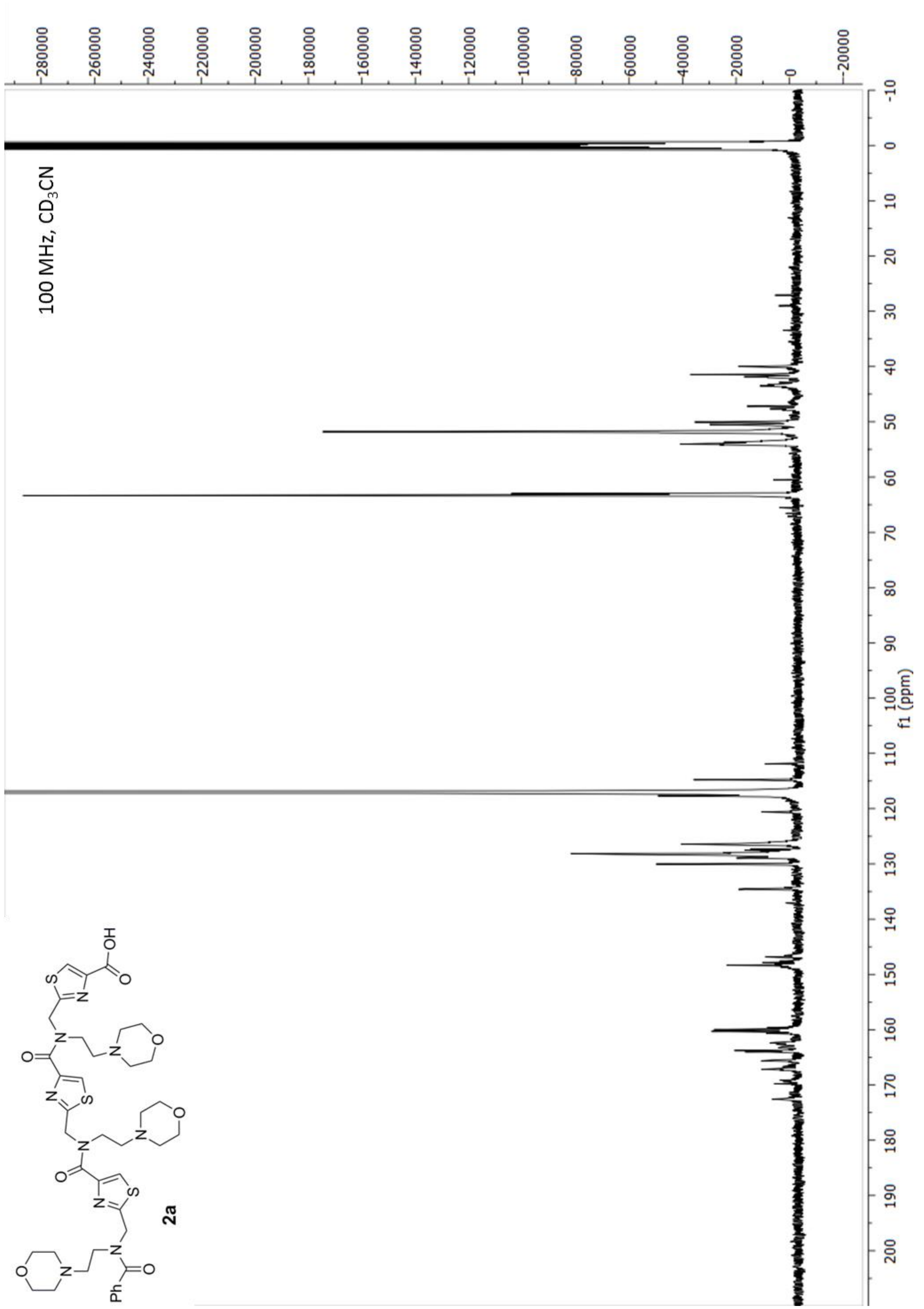
100 MHz, CD3CN
f1 (ppm)
2a

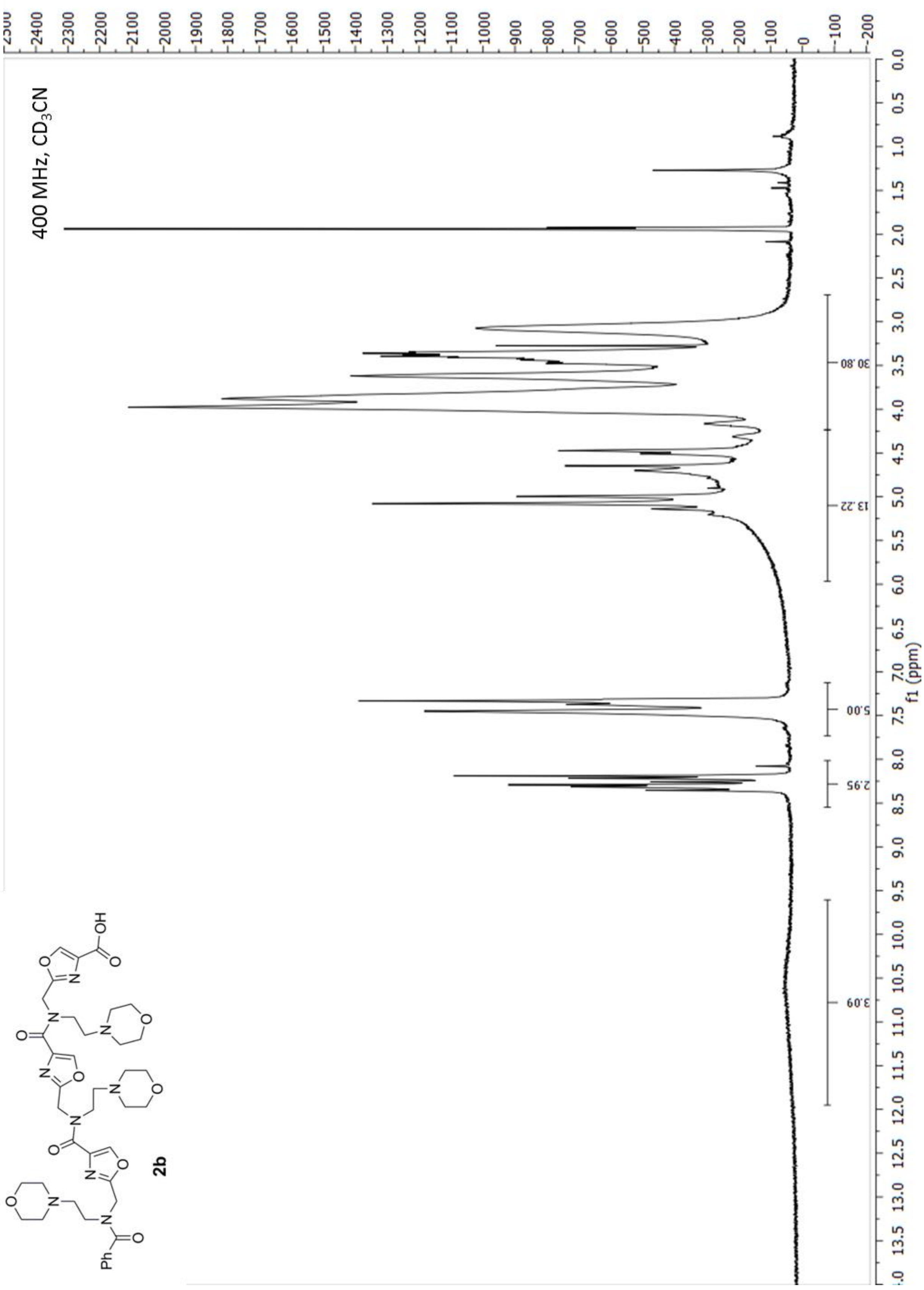
400 MHz, CD3CN
2b
f1 (ppm)
30.80
13.22
5.00
2.95
3.09

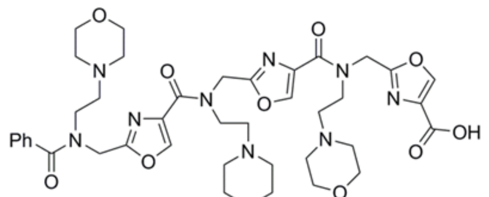

2b

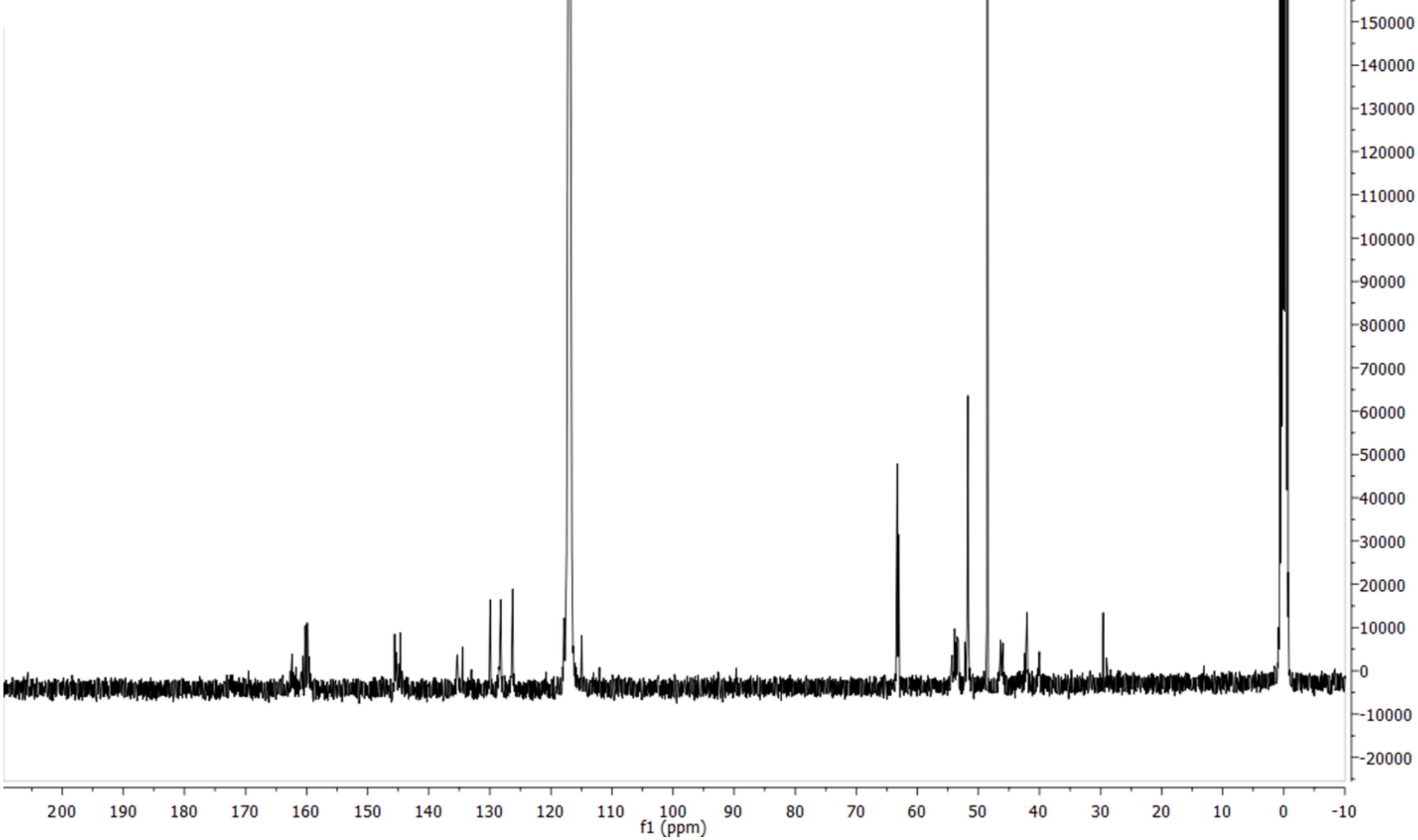

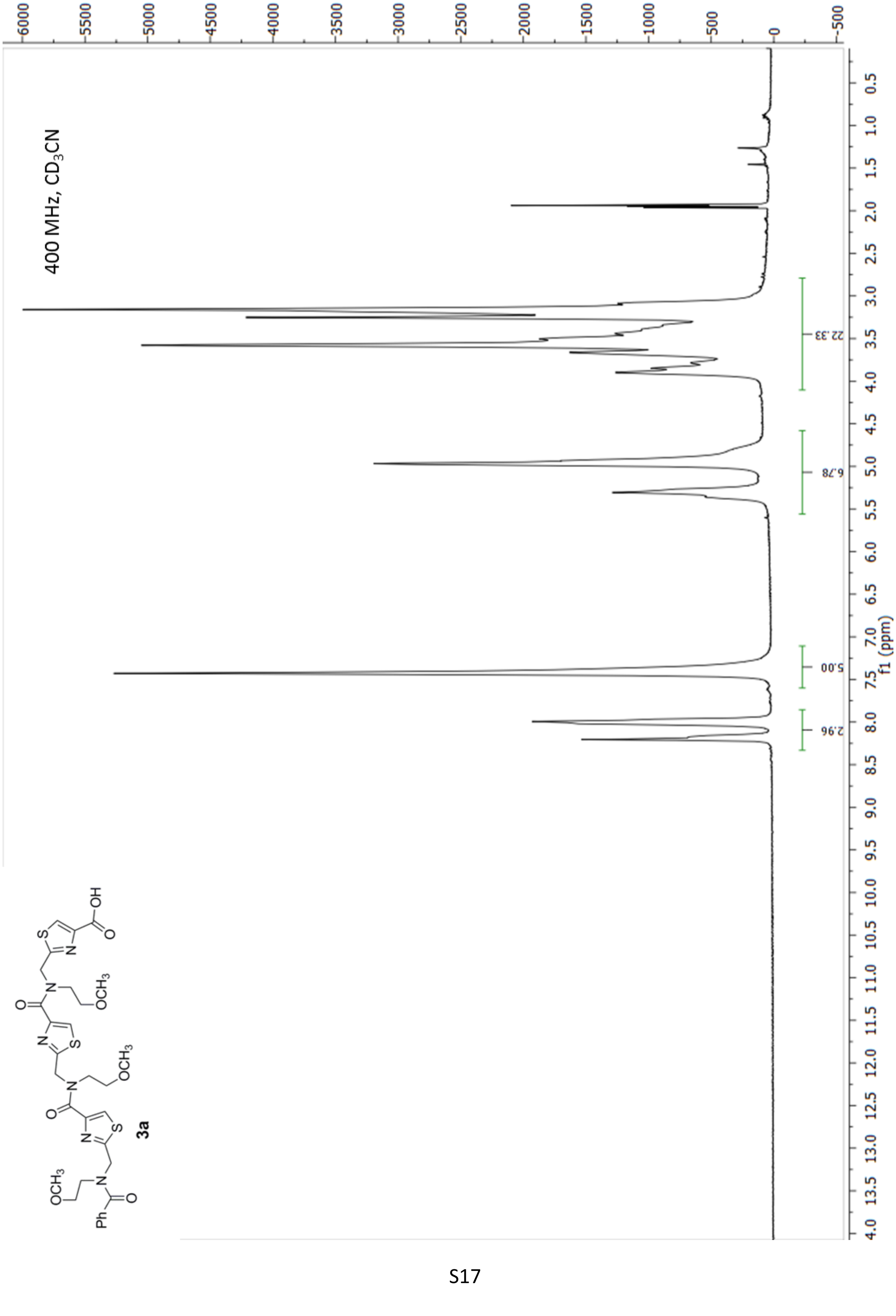
400 MHz, CD3CN
f1 (ppm)
3a

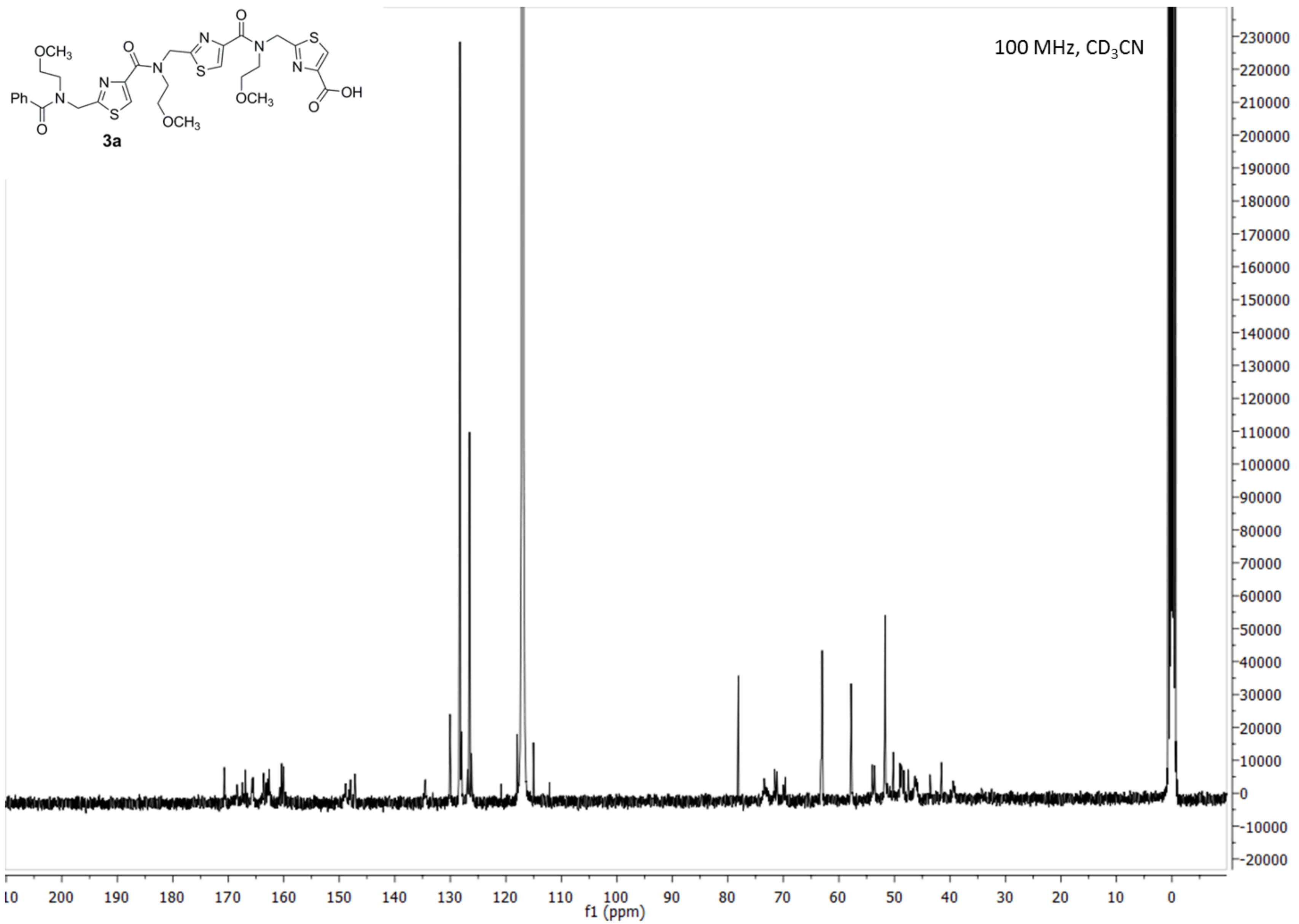

100 MHz, CD_3CN
3a
f1 (ppm)

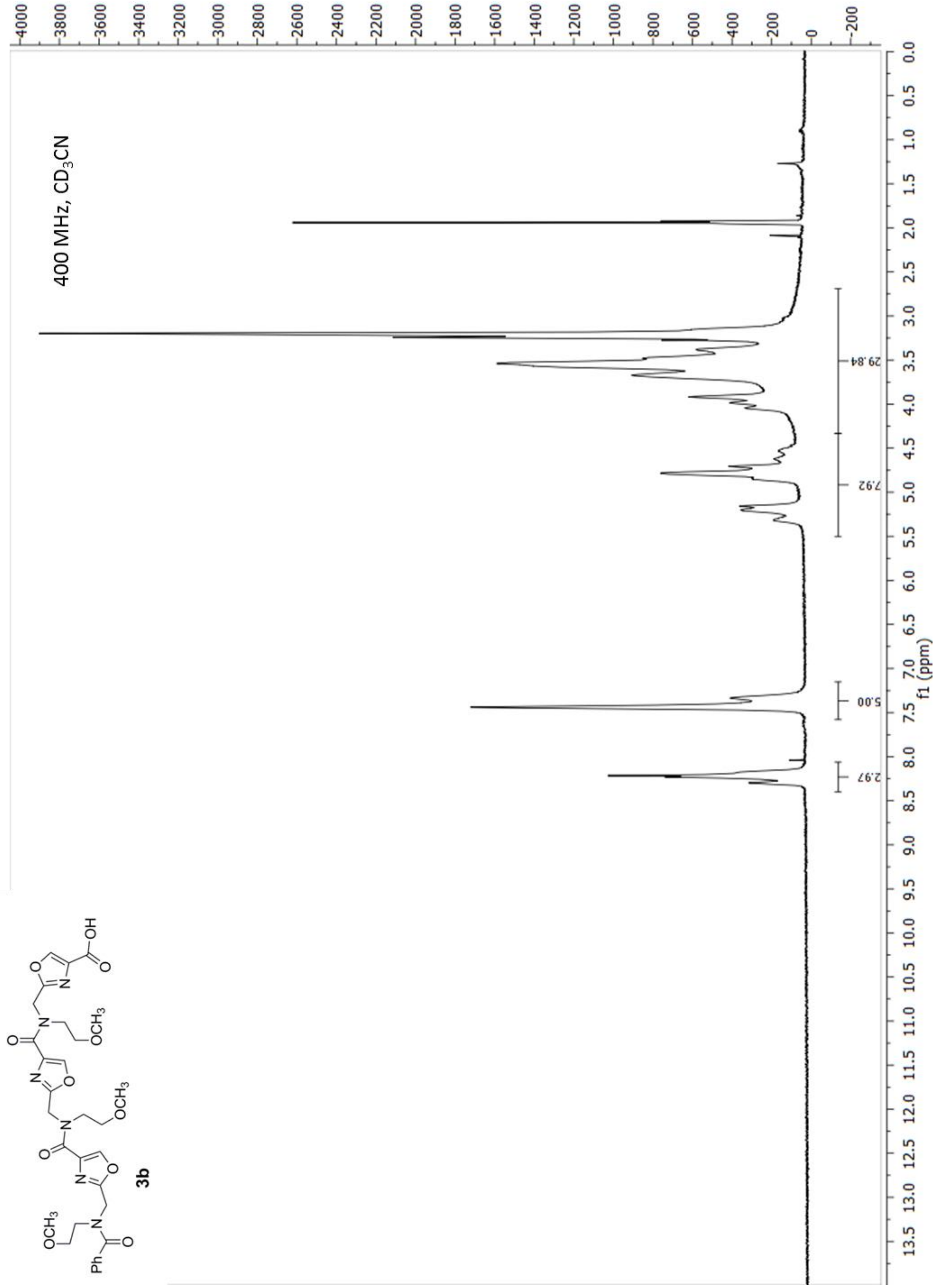
400 MHz, CD3CN
29.84
7.92
5.00
2.97
f1 (ppm)
3b

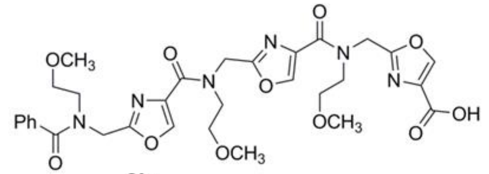
OCH3
Ph
N
O
N
O
O
N
OCH3
N
O
O
N
OCH3
N
O
OH
O

3b

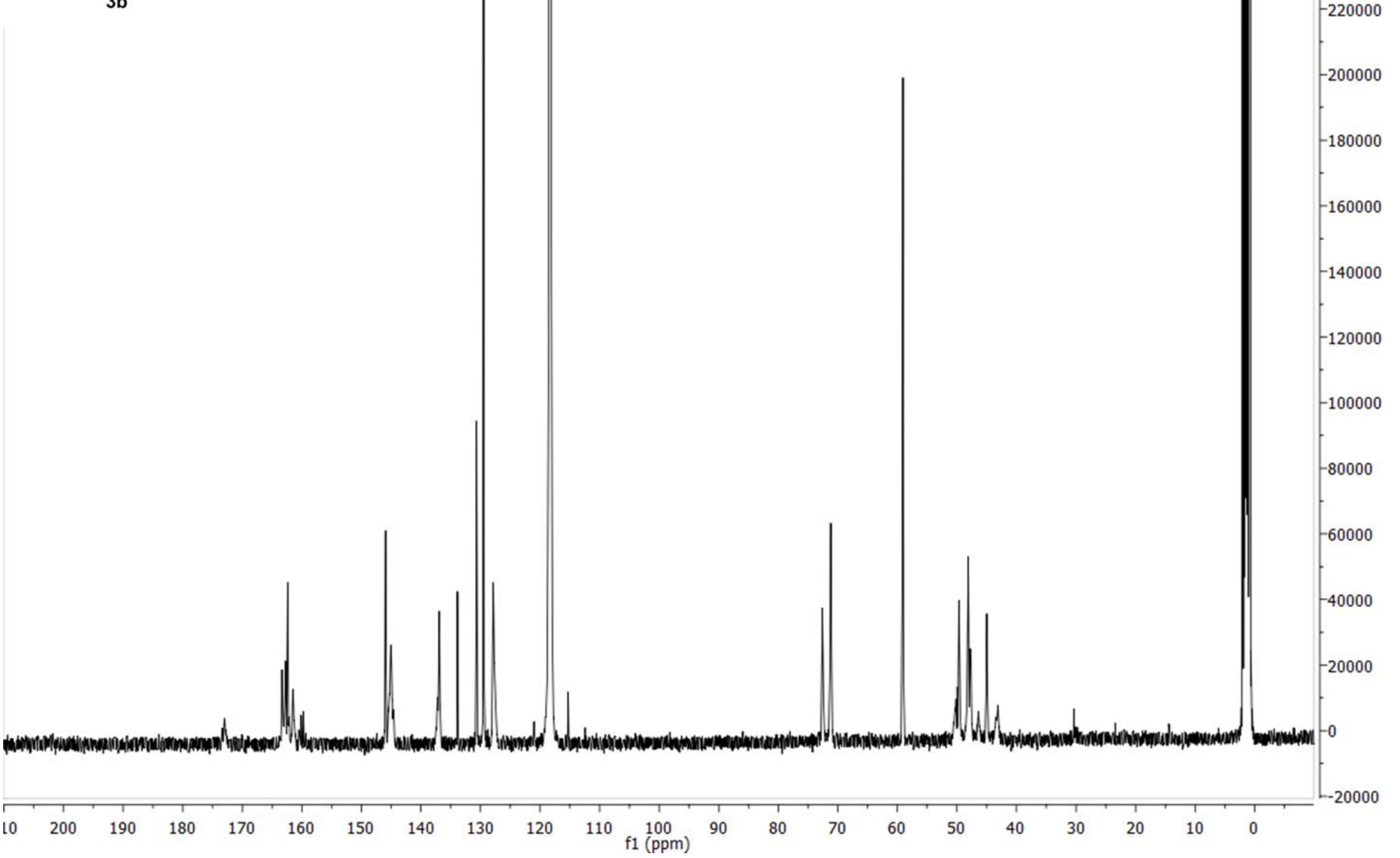
100 MHz, CD3CN
f1 (ppm)
10
200
190
180
170
160
150
140
130
120
110
100
90
80
70
60
50
40
30
20
10
0
260000
240000
220000
200000
180000
160000
140000
120000
100000
80000
60000
40000
20000
0
-20000

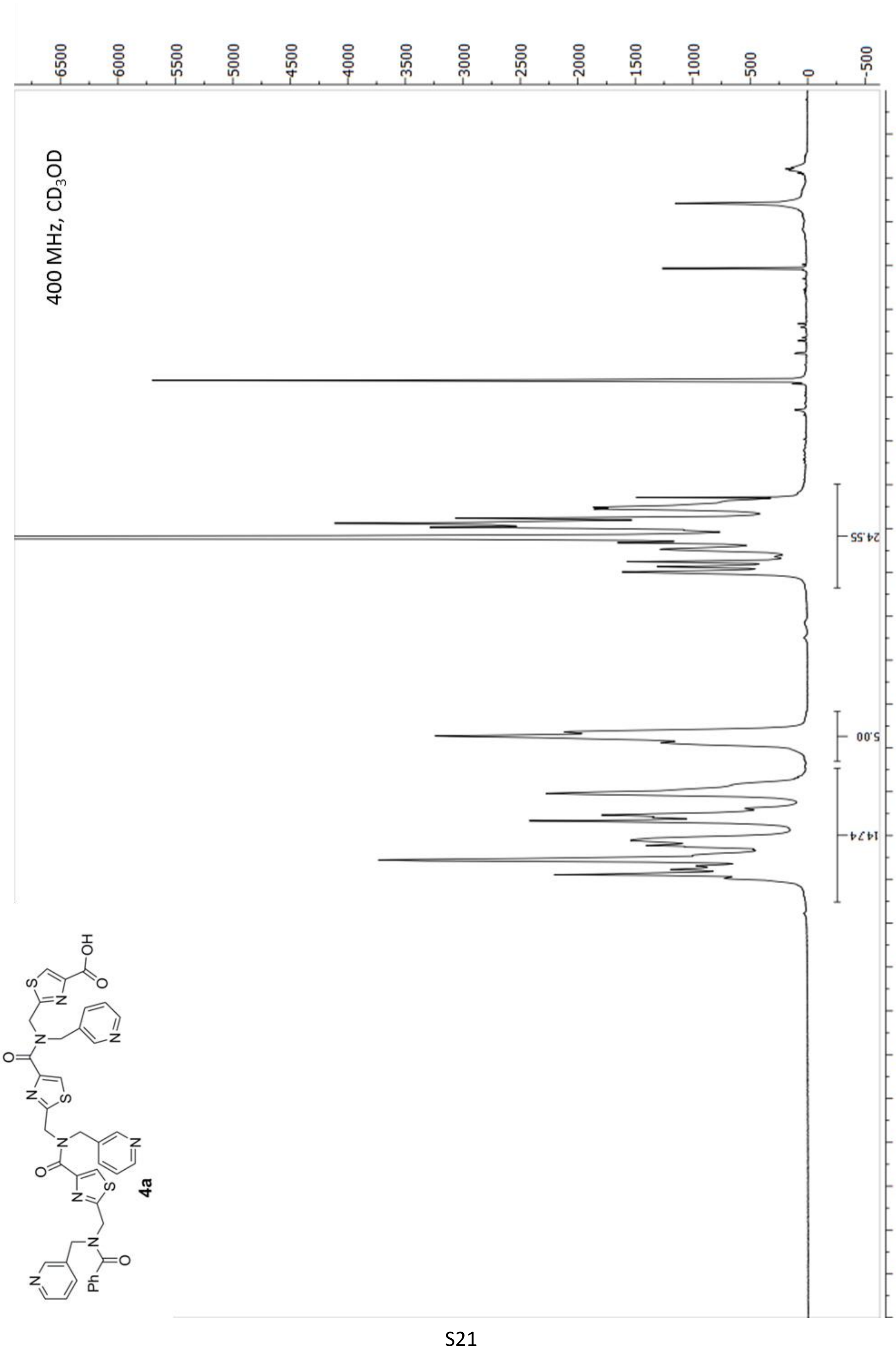

4a

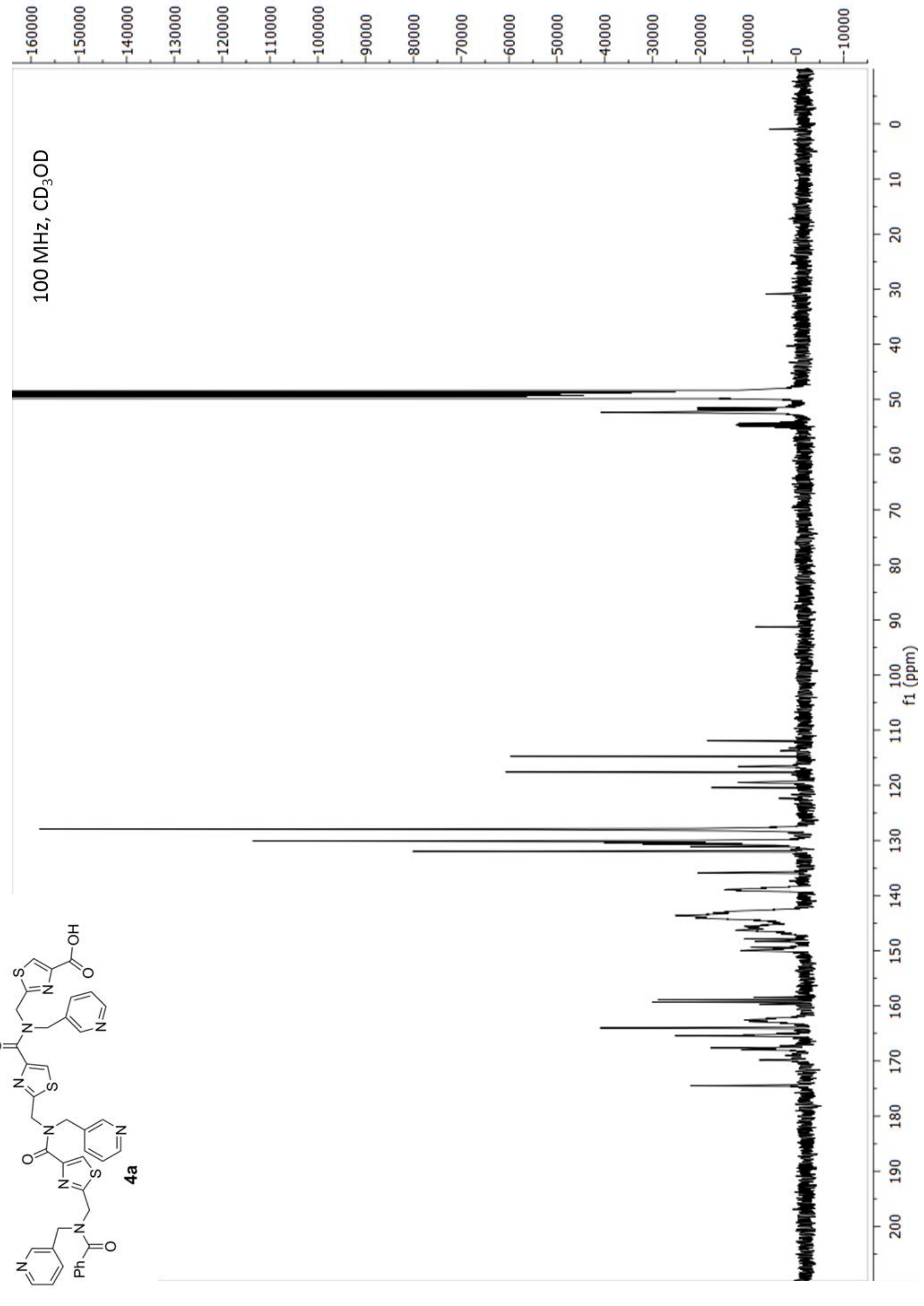
100 MHz, CD_3OD
f1 (ppm)
4a

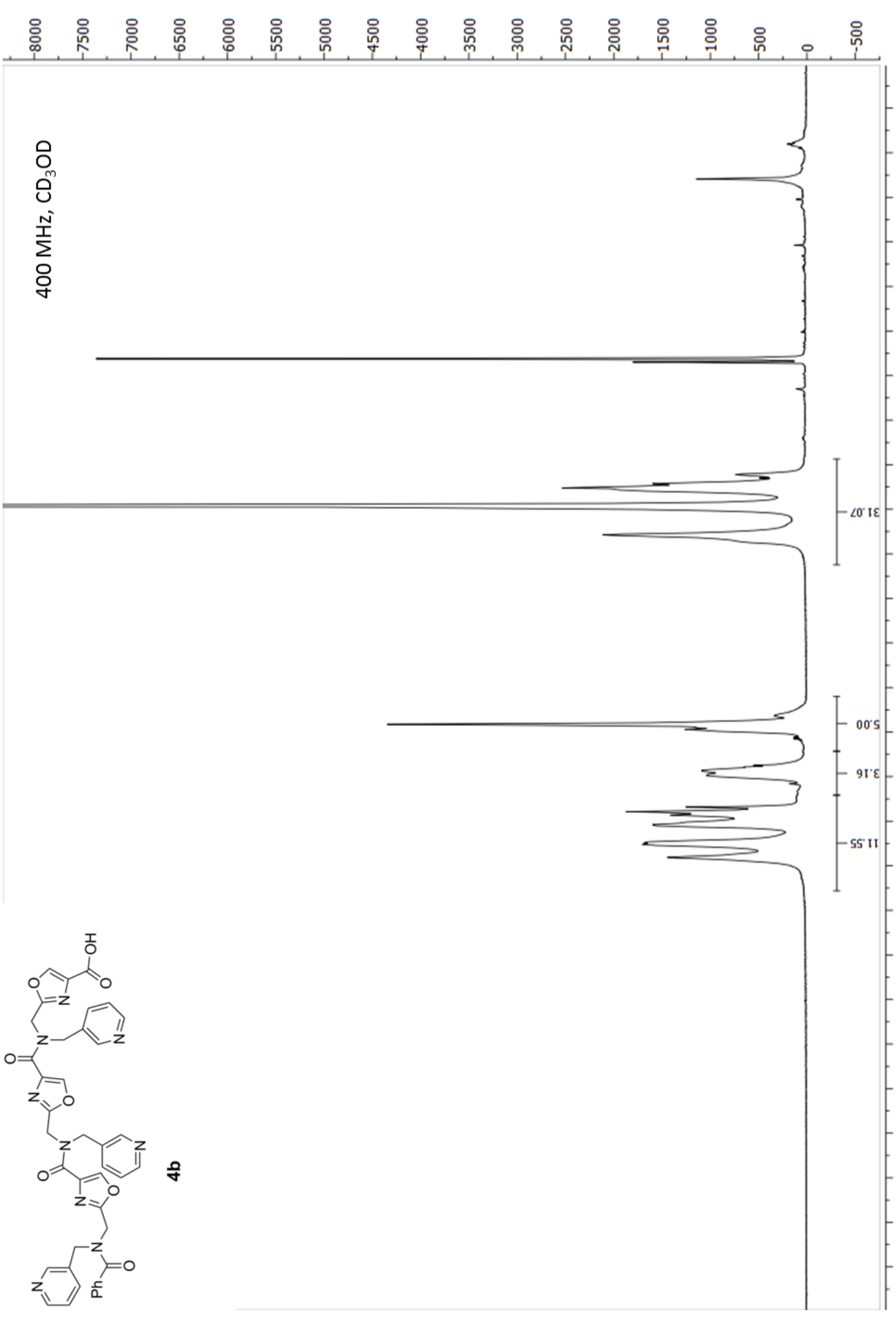
400 MHz, CD_3OD
f1 (ppm)
4b

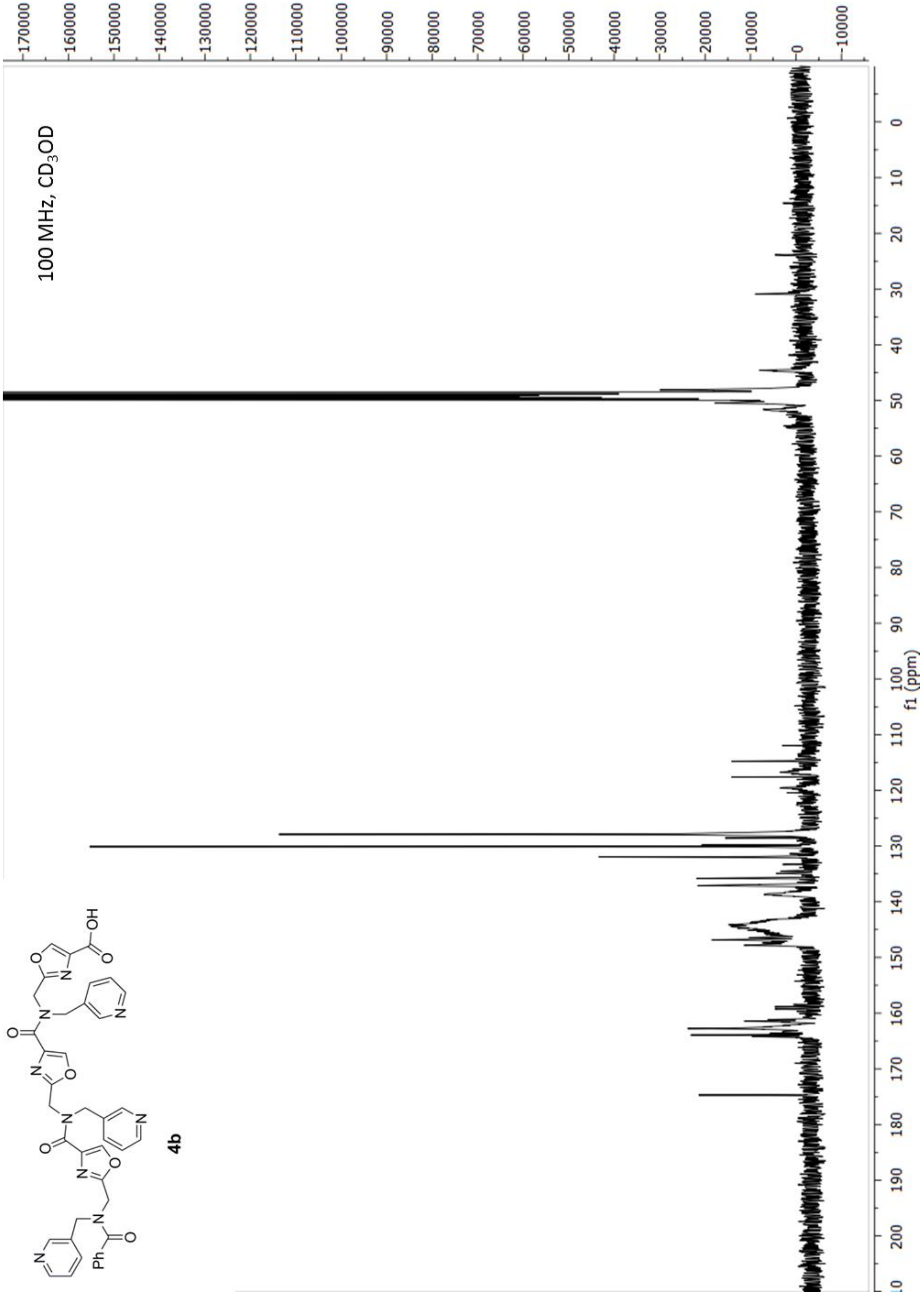
100 MHz, CD$_3$OD
4b
f1 (ppm)

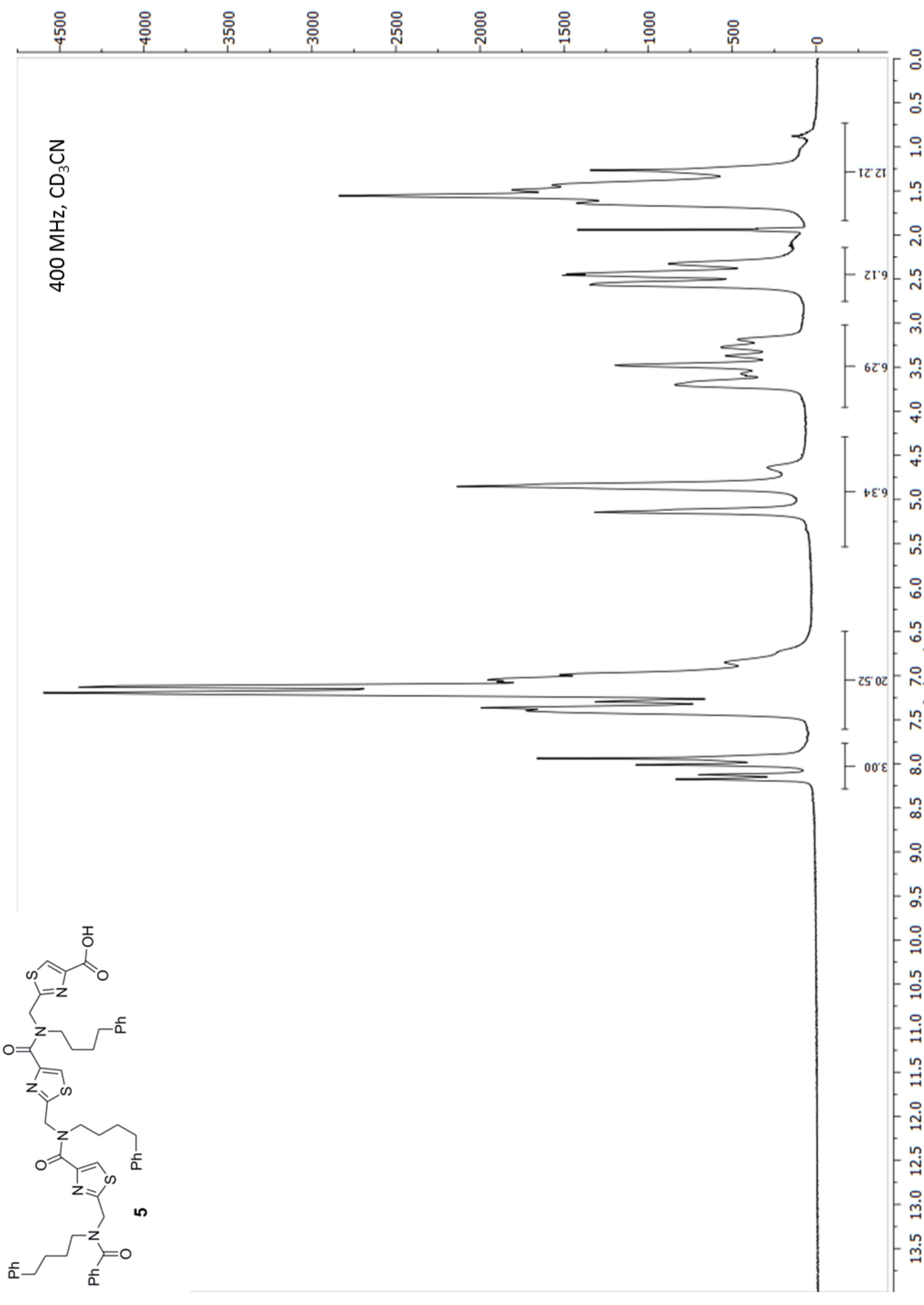
400 MHz, CD3CN
f1 (ppm)
5

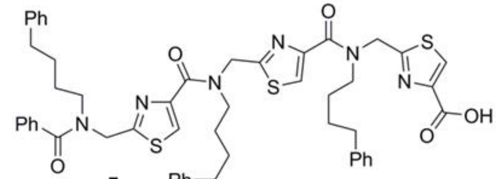

5

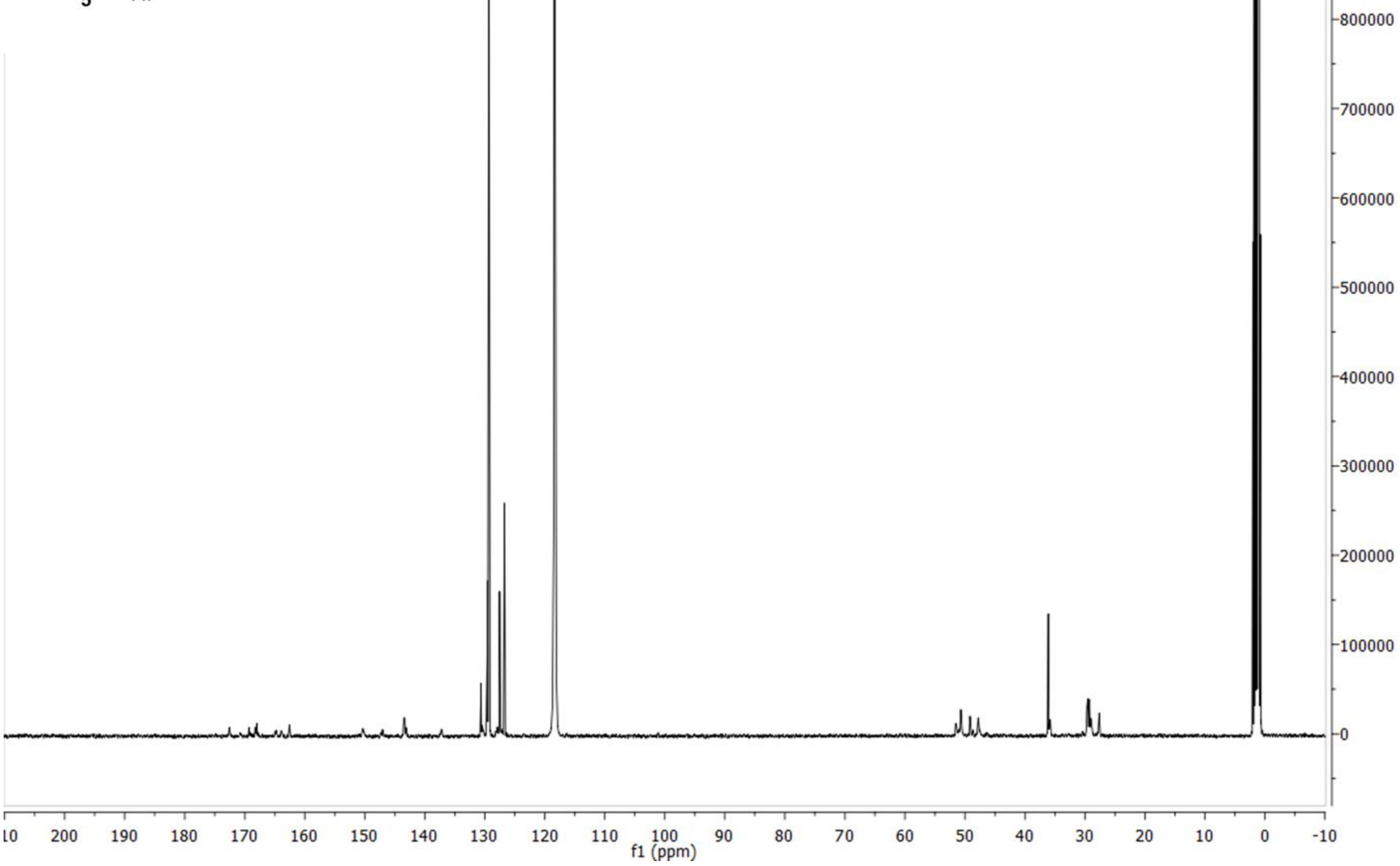

100 MHz, CD$_3$CN
900000
800000
700000
600000
500000
400000
300000
200000
100000
0
10
200
190
180
170
160
150
140
130
120
110
100
90
80
70
60
50
40
30
20
10
0
-10
f1 (ppm)

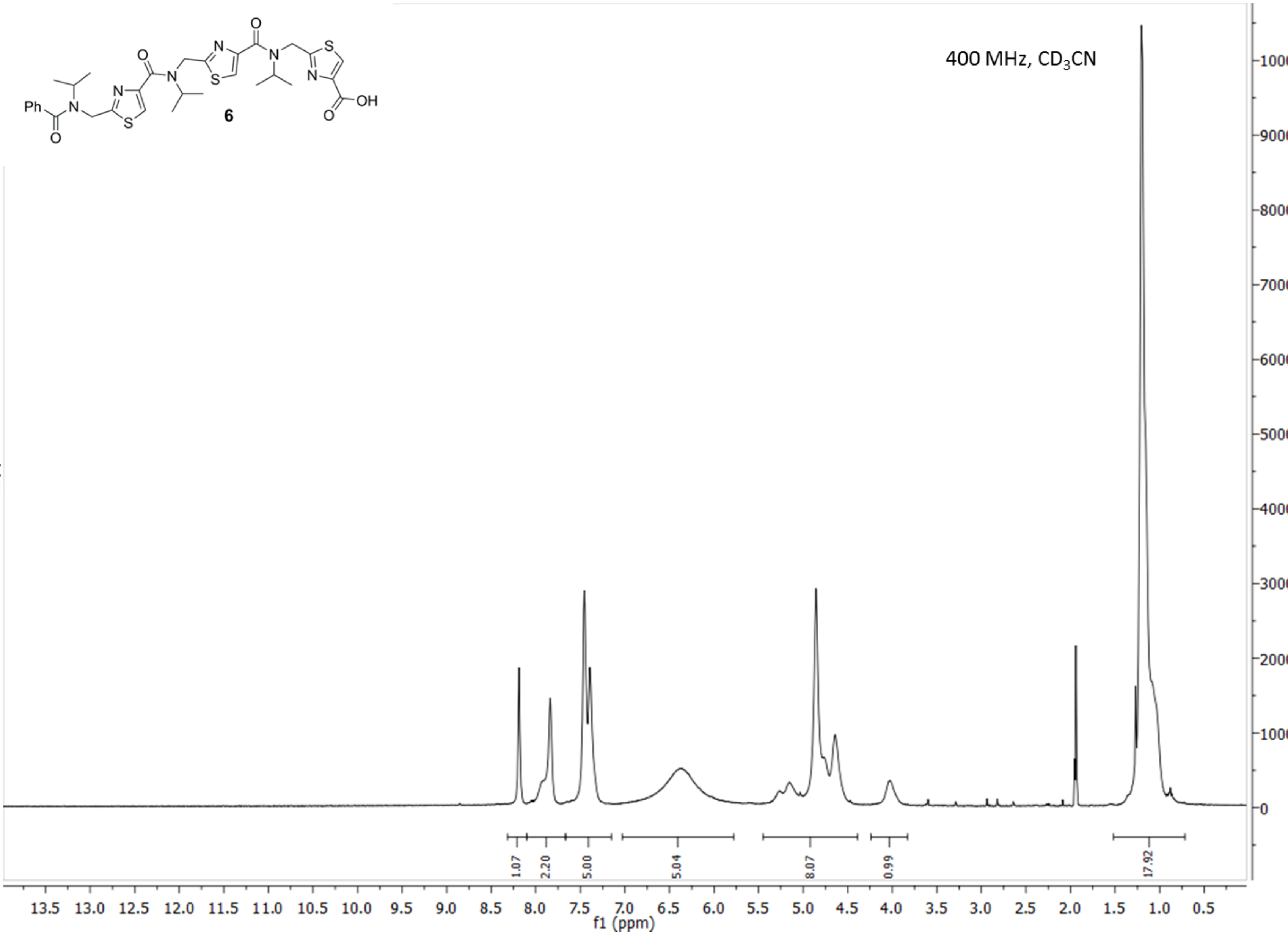
6
400 MHz, CD_3CN
1.07
2.20
5.00
5.04
8.07
0.99
17.92
f1 (ppm)

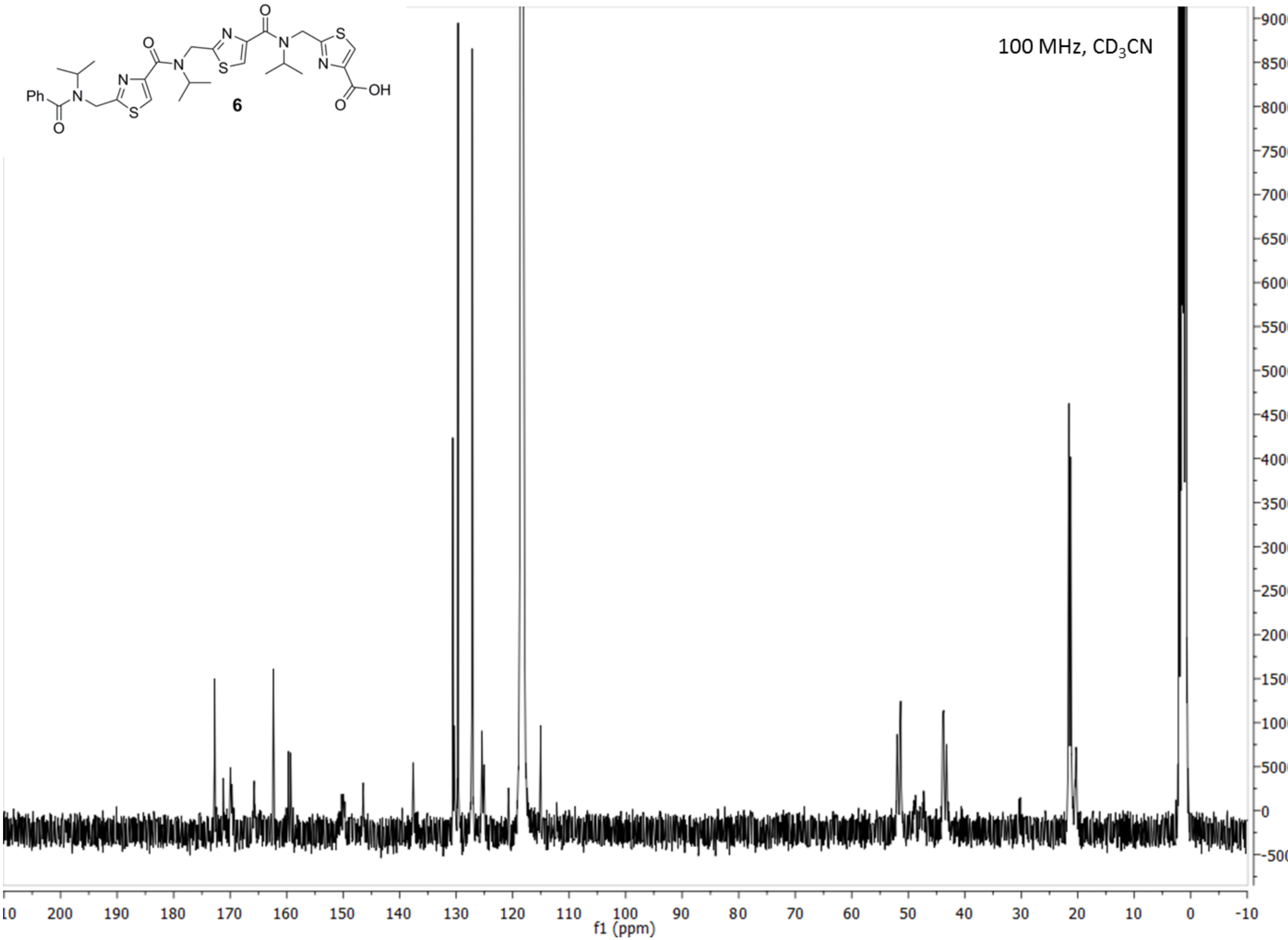
100 MHz, CD_3CN
6
f1 (ppm)

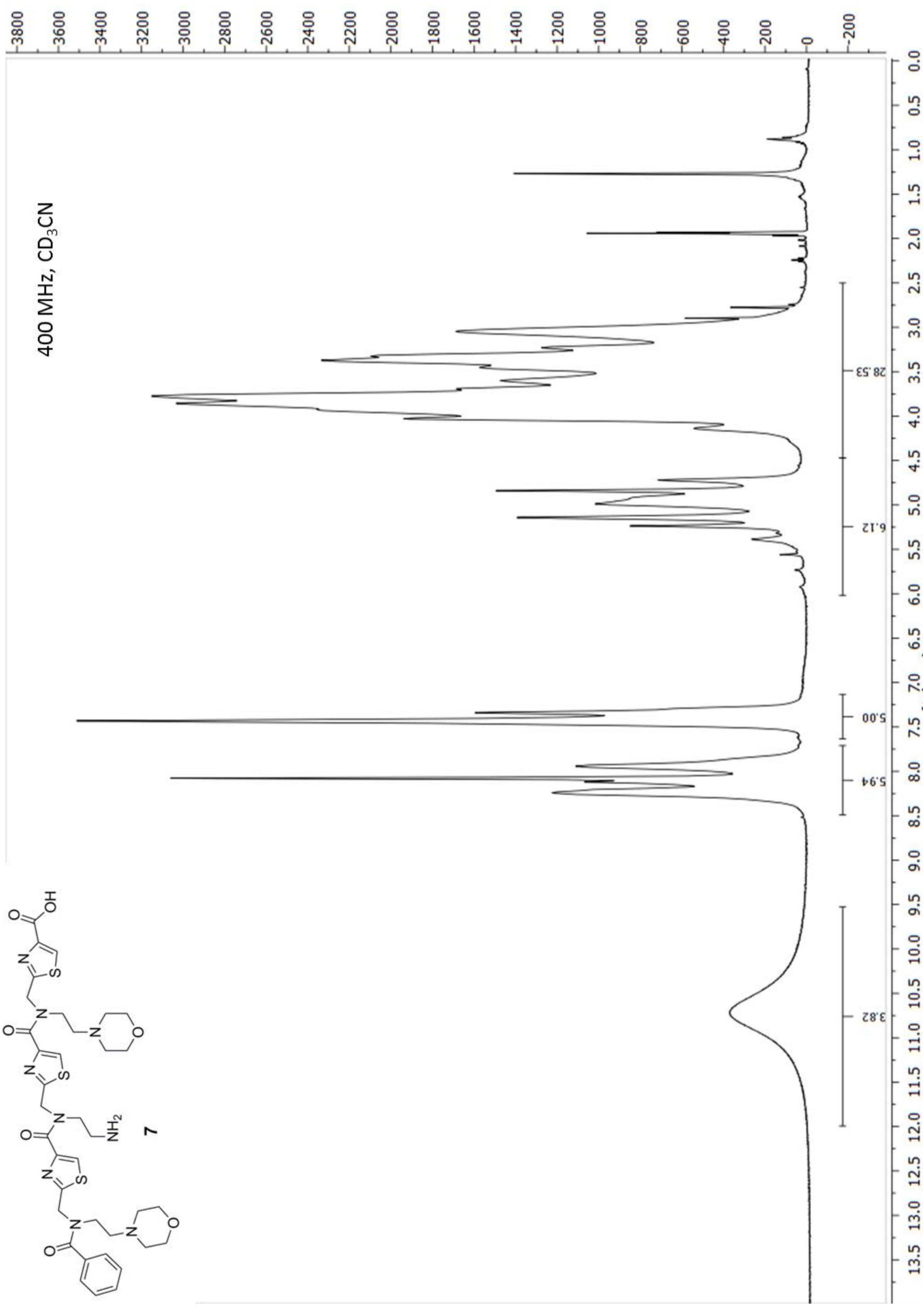
400 MHz, CD3CN
7
28.53
6.12
5.00
5.94
3.82
f1 (ppm)

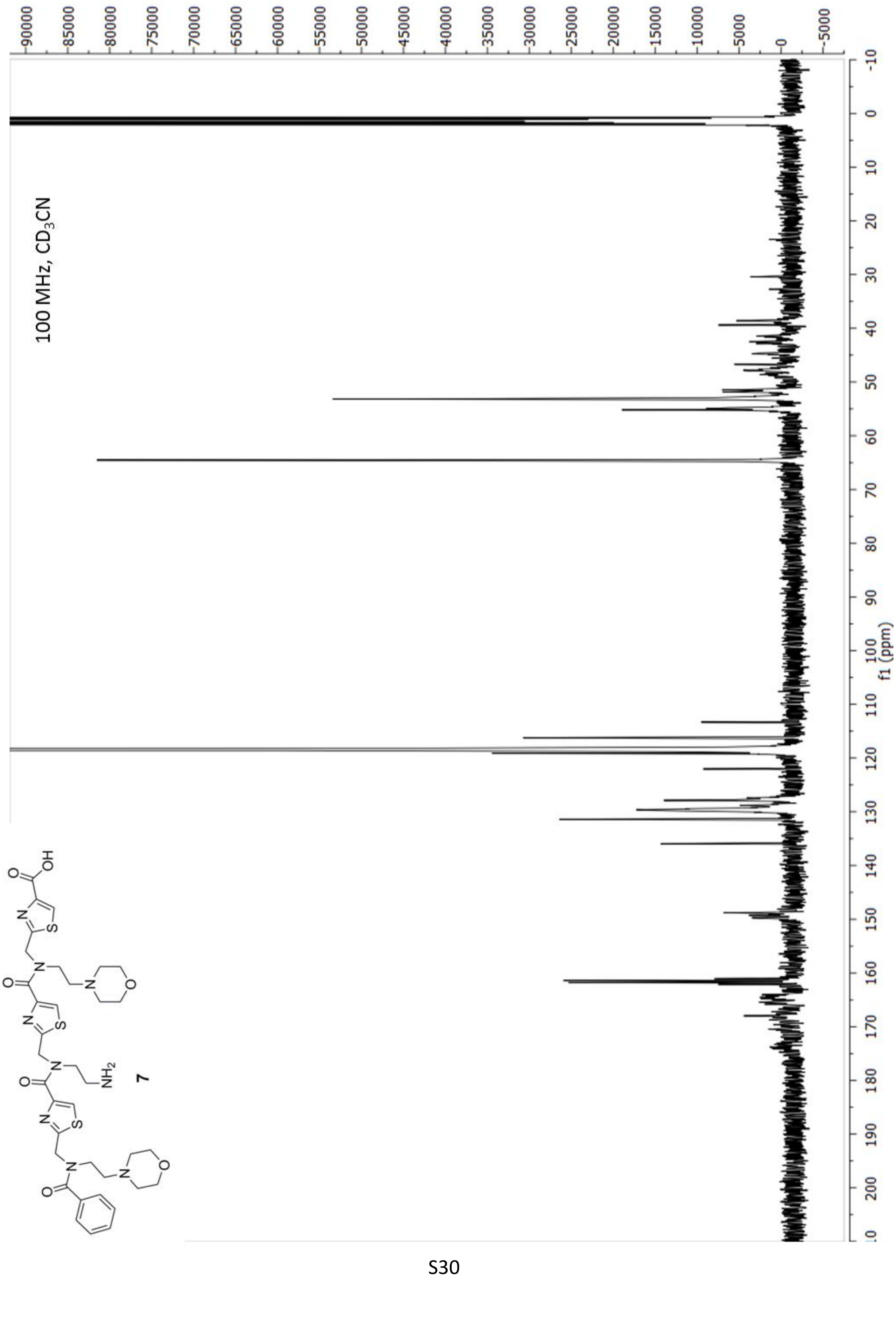
100 MHz, CD3CN
f1 (ppm)
7

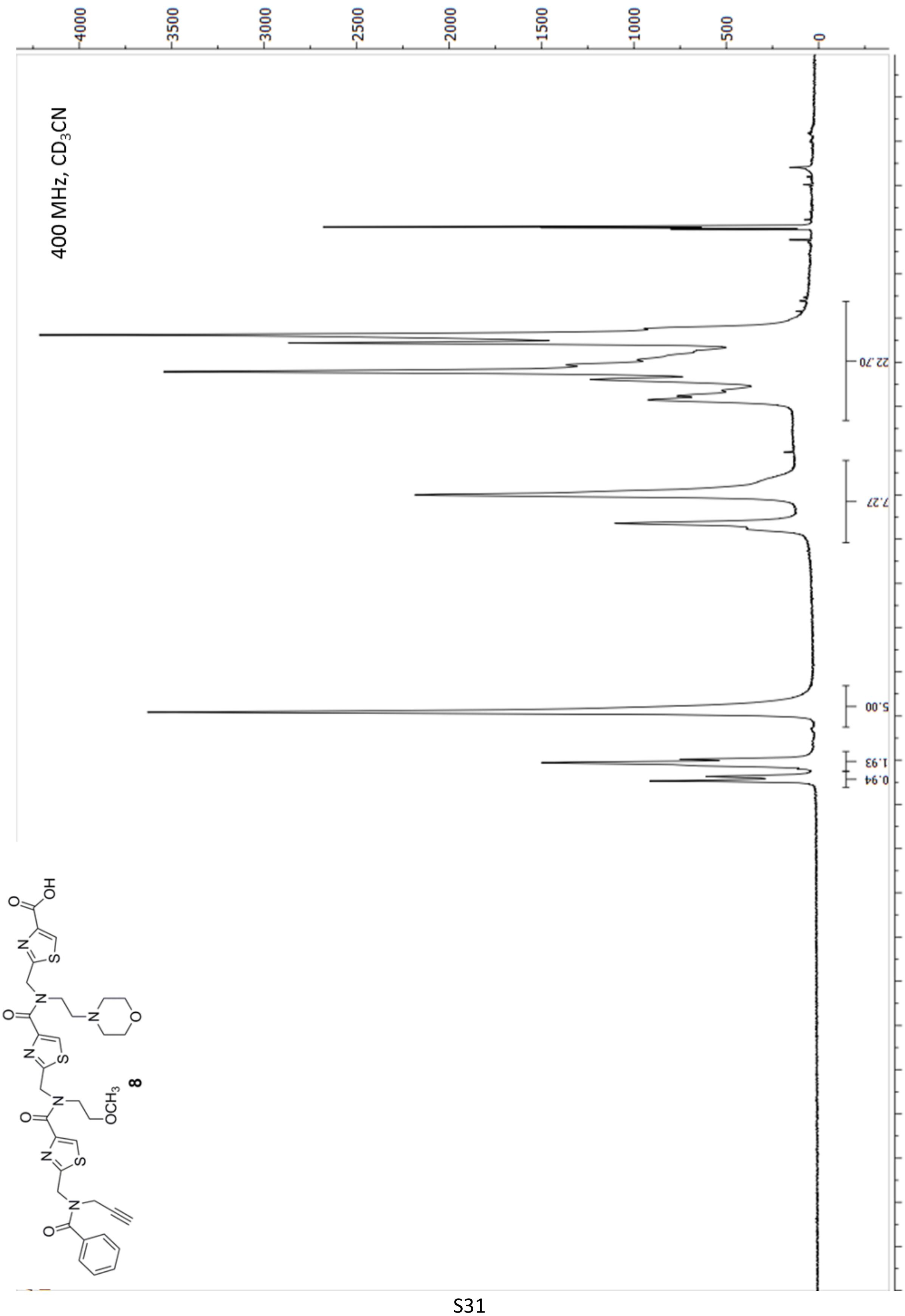
400 MHz, CD3CN
8
f1 (ppm)
22.70
7.27
5.00
1.93
0.94

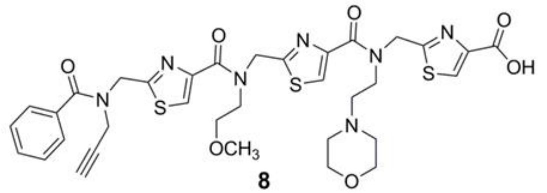

8

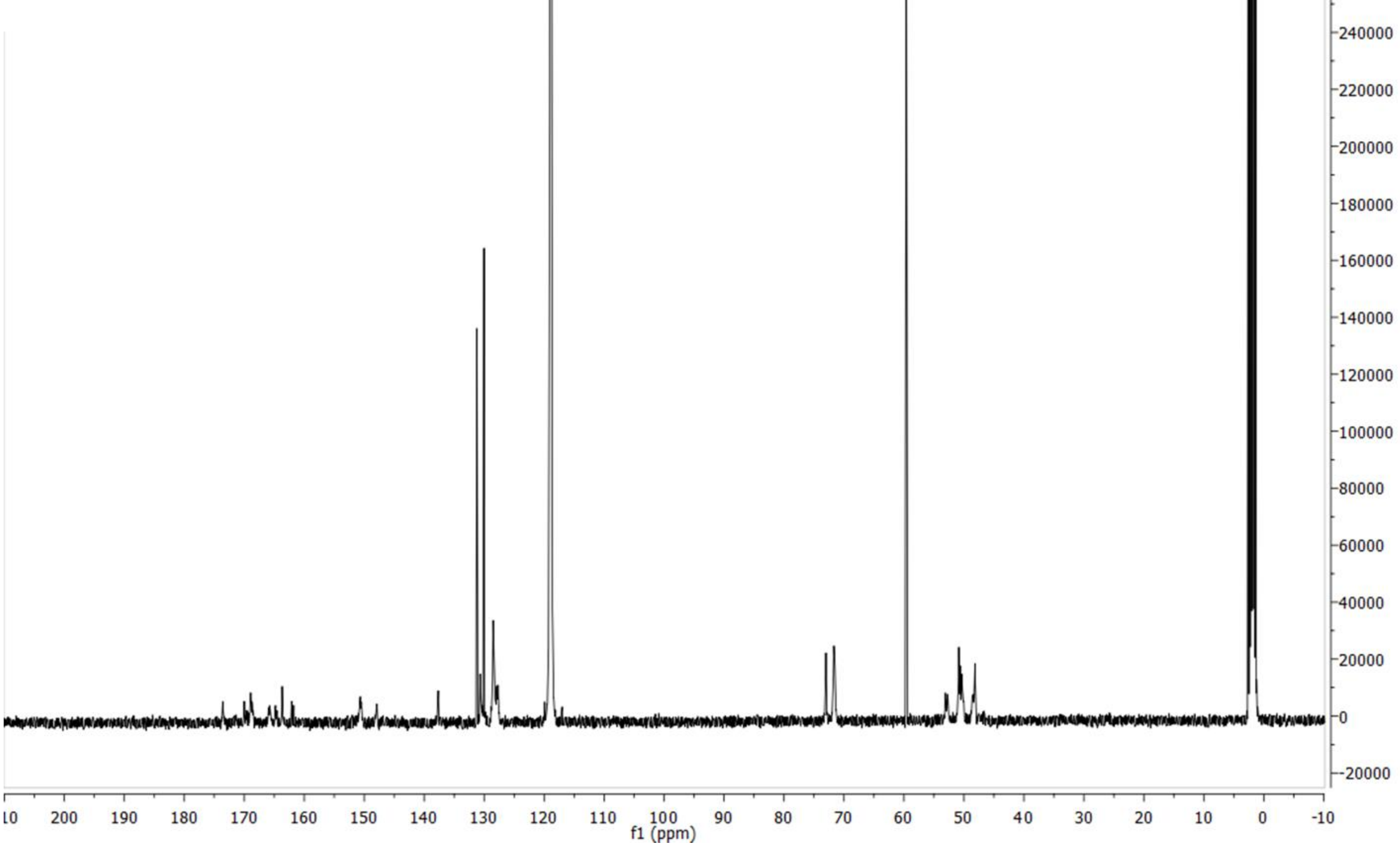

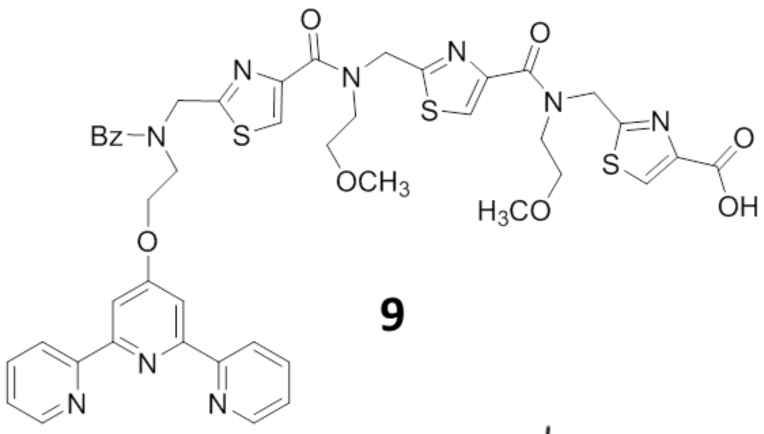
Bz
N
O
S
OCH3
H3CO
OH

9

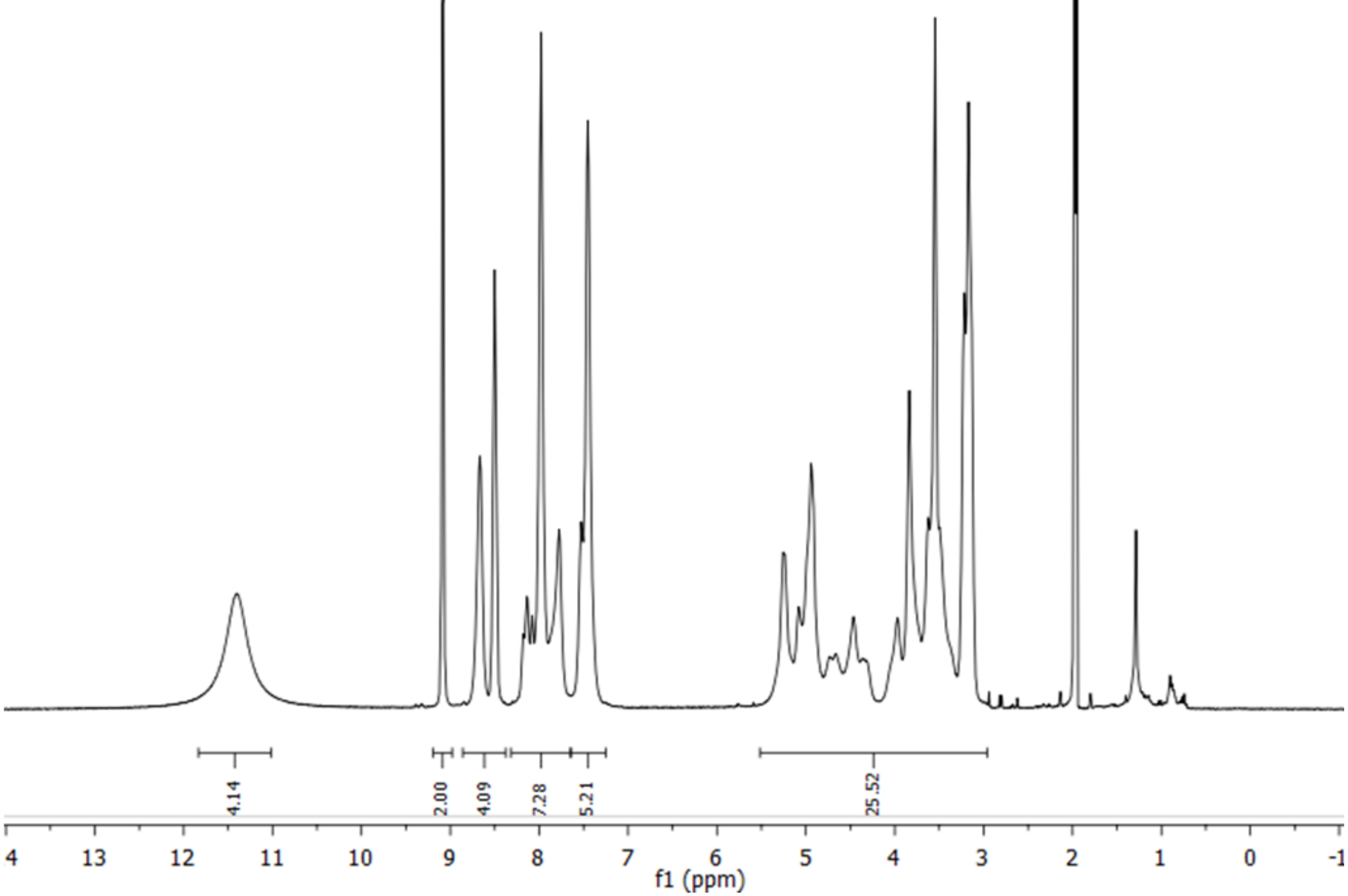
400 MHz, CD3CN
4.14
2.00
4.09
7.28
5.21
25.52
4
13
12
11
10
9
8
7
6
5
4
3
2
1
0
-1
f1 (ppm)

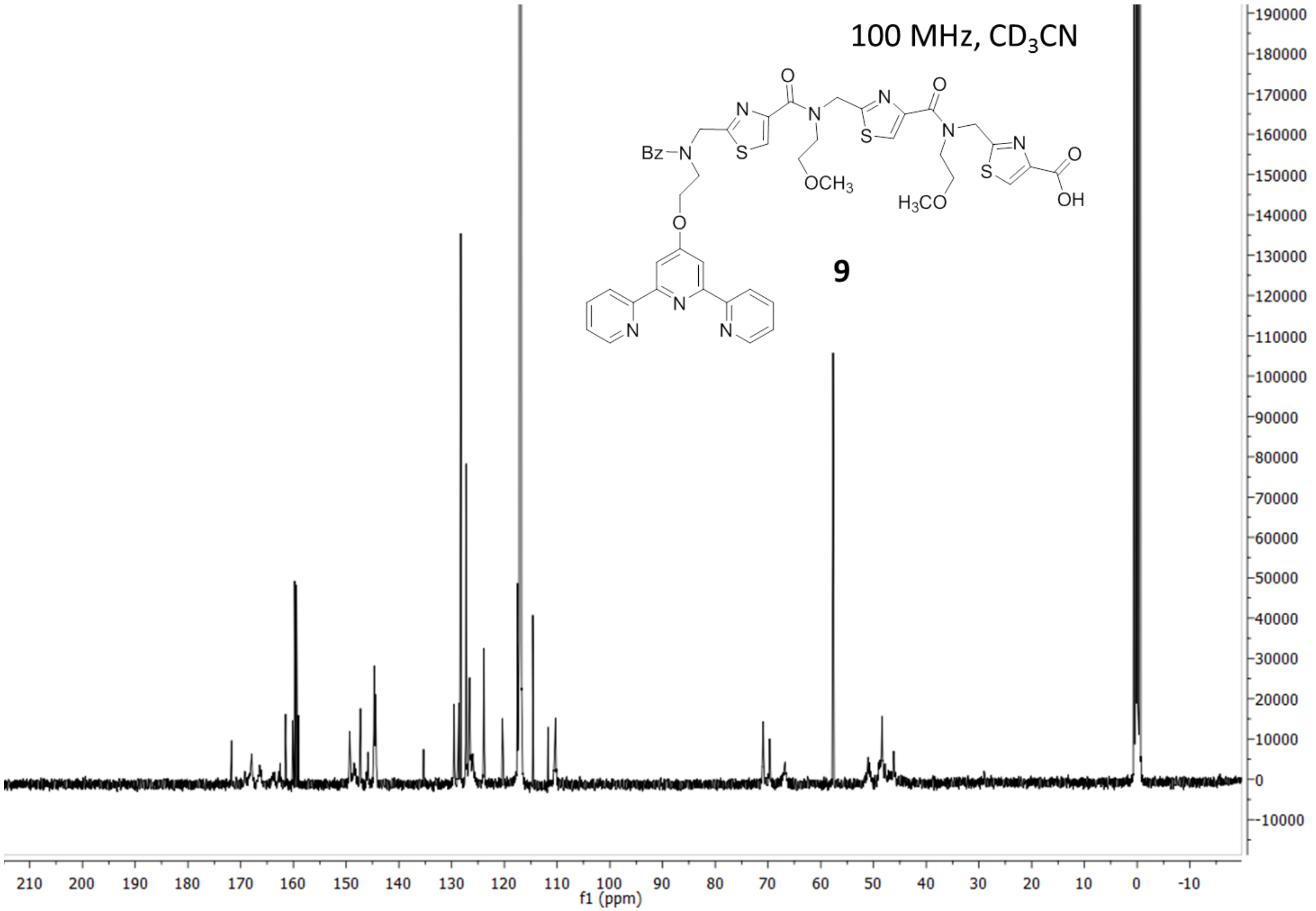
100 MHz, CD_3CN
9
Bz
N
S
O
OCH_3
H_3CO
OH
f1 (ppm)

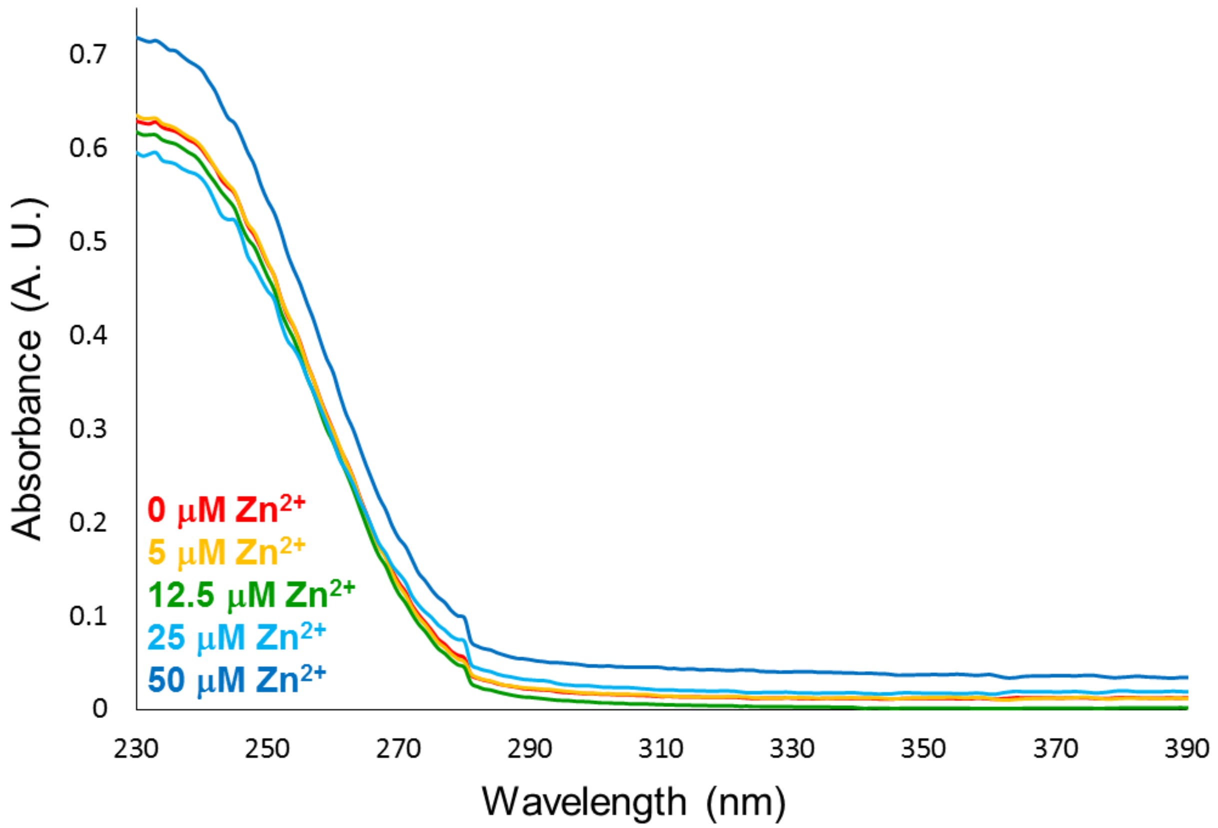

Figure S4. UV spectrum of 50 μM **3a** in the presence of increasing $ZnCl_2$ in 10 mM Tris buffer, pH 7.5.

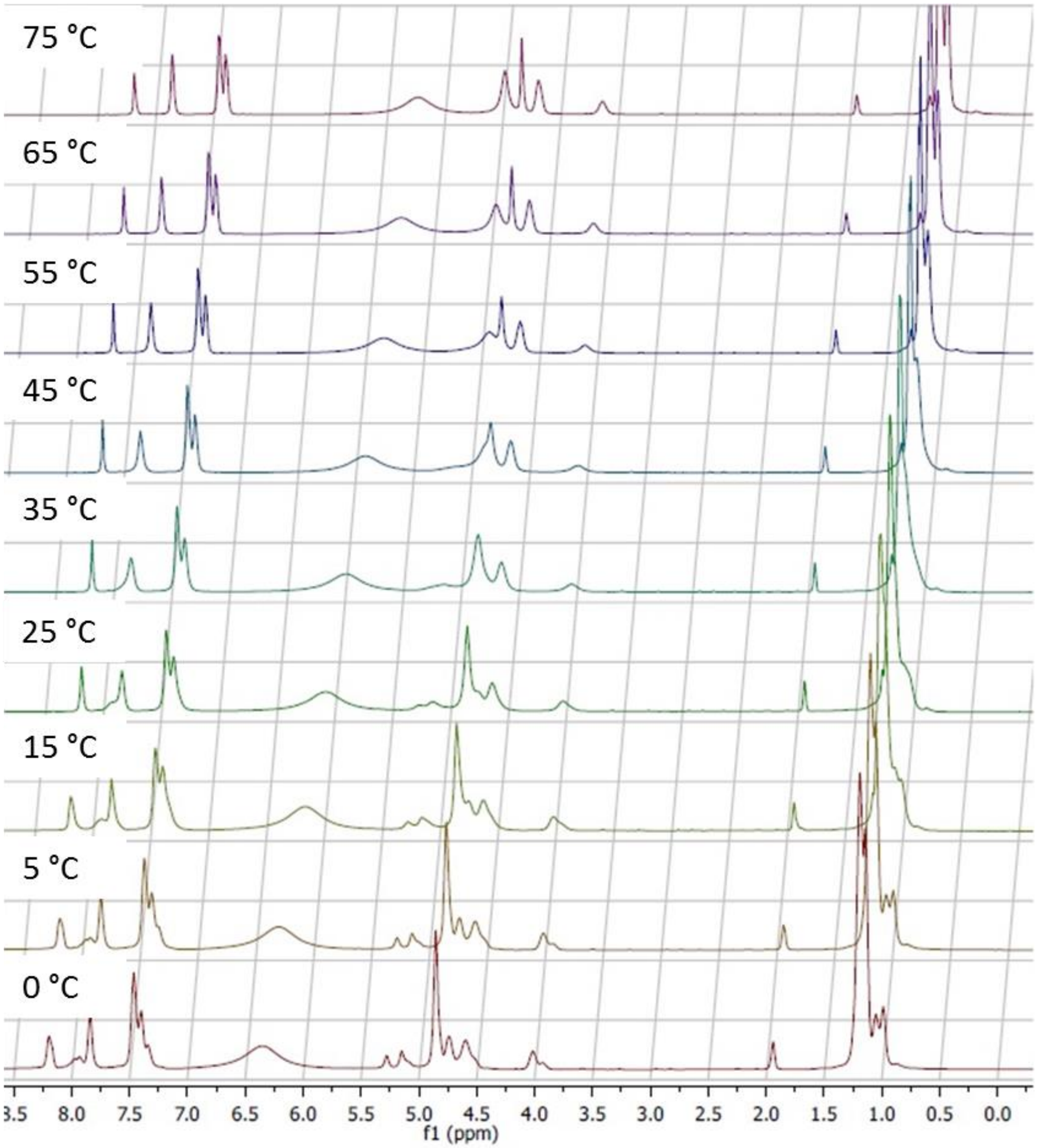

Figure S5. ^{1}H NMR temperature study of **6** in CD_3CN shows evidence of azole peptoid flexibility.
